# ProteoAnalyst: Connecting biological context and peptide‐level evidence in quantitative proteomics and phosphoproteomics

**DOI:** 10.1002/pro.70851

**Published:** 2026-11-12

**Authors:** Guangyan Zhou, Gordon Li, Danielle M. Simons, Alyssa Weinrauch, Nolan Sammut, Jelena Kolic, Haoning Howard Cen, Daina Avizonis, Peter Siegel, Francis C. Lynn, Patrick E. MacDonald, James D. Johnson, Jean‐François Trempe, Jianguo Xia

**Affiliations:** ^1^ Department of Microbiology and Immunology McGill University Montreal Quebec Canada; ^2^ Institute of Parasitology McGill University Montreal Quebec Canada; ^3^ Department of Pharmacology and Therapeutics and Centre de Recherche en Biologie Structurale McGill University Montreal Quebec Canada; ^4^ Life Sciences Institute, Department of Cellular and Physiological Sciences & Department of Surgery University of British Columbia Vancouver British Columbia Canada; ^5^ Department of Surgery University of British Columbia Vancouver British Columbia Canada; ^6^ Department of Physiology University of Alberta Edmonton Alberta Canada; ^7^ Department of Biochemistry McGill University Montreal Quebec Canada; ^8^ Rosalind and Morris Goodman Cancer Institute McGill University Montreal Quebec Canada; ^9^ Diabetes Research Group BC Children's Hospital Research Institute Vancouver British Columbia Canada; ^10^ Department of Pharmacology, Faculty of Medicine and Dentistry University of Alberta Edmonton Alberta Canada; ^11^ Alberta Diabetes Institute University of Alberta Edmonton Alberta Canada; ^12^ Brain Repair and Integrative Neuroscience (BRaIN) Research Institute of the McGill University Health Centre (RI‐MUHC) Montréal Québec Canada

**Keywords:** data‐independent acquisition, differential abundance analysis, mass spectrometry, pathway enrichment, phosphoproteomics, quantitative proteomics, subcellular localization

## Abstract

Modern mass spectrometry now routinely quantifies thousands of proteins and phosphosites. While raw spectral processing and quantification have matured over the past few years, downstream statistical analysis and functional interpretation remain challenging. Current tools are often fragmented across separate applications for statistics, enrichment, network analysis, and phosphoproteomics. Furthermore, they operate largely at the protein or site level, leaving the supporting peptide, precursor, and phosphosite measurements out of reach; while pathway and network visualizations are provided without subcellular context. To address these limitations, we developed ProteoAnalyst, an easy‐to‐use web‐based platform that takes common upstream outputs (MaxQuant, FragPipe, DIA‐NN, and Spectronaut) through quality control, statistical analysis, and functional interpretation within a single environment. Researchers can navigate from pathways, compartment‐aware interaction networks, and kinase‐substrate networks down to the individual proteins, peptides, precursors, and phosphosites that support them. Dedicated modules extend this capacity to peptide‐level detection of isoform usage and post‐translational modification (PTM) relative fractions, phosphoproteomics with protein abundance correction, and biomarker analysis. Our benchmarks and case studies demonstrate that ProteoAnalyst integrates well‐established methods with rich functional analysis and visualization to streamline data interpretation and hypothesis generation. ProteoAnalyst is freely available at www.proteoanalyst.ca.

## 1 INTRODUCTION

As mass spectrometry (MS)‐based bottom‐up proteomics advances, the volume and complexity of generated data are expanding rapidly. Data‐independent acquisition (DIA) now routinely quantifies many thousands of proteins per run, approaching comprehensive coverage of mammalian proteomes, and the same peptide‐level data also support high‐throughput profiling of post‐translational modifications (PTMs) such as phosphorylation. The upstream processing of raw LC–MS/MS data into quantitative peptide and protein abundance tables is now well supported by software suites such as MaxQuant (Cox & Mann, 2008), FragPipe (Kong et al., 2017), DIA‐NN (Demichev et al., 2020), and Spectronaut (Bruderer et al., 2015), which handle feature detection, peptide‐spectrum matching, false discovery rate (FDR) estimation, and protein inference. Consequently, downstream data analysis and interpretation have become the primary analytical bottleneck. Users must still assess data quality, choose appropriate filtering, normalization, and missing‐value strategies, perform statistical comparisons, and convert lists of altered proteins, peptides, or phosphosites into biologically interpretable hypotheses.

A range of bioinformatics platforms currently support downstream proteomics analysis. Perseus (Tyanova et al., 2016) provides a comprehensive desktop environment for matrix‐based normalization, clustering, statistical testing, and enrichment. Several dedicated statistical frameworks provide rigorous quantification: MSstats (Kohler, Staniak, et al., 2023) for model‐based differential testing, DEqMS (Zhu et al., 2020) for peptide‐count‐based variance estimation, and AlphaPeptStats (Krismer et al., 2023) for automated Python‐based workflows. Web‐based platforms have broadened accessibility: the Analyst Suite (Shah et al., 2019; Zhang et al., 2023) from Monash Proteomics offers workflow‐specific applications for label‐free, DIA, TMT, and phosphoproteomics data, with a separate Pathway‐Analyst tool for enrichment and network analysis; amica (Didusch et al., 2022) supports quality control, differential expression, and Gene Ontology enrichment; and ProteoArk (Nisar et al., 2025) combines preprocessing, differential expression, functional enrichment, and network visualization in a single interface. A feature‐level comparison of these representative tools is summarized in Table S1. Despite these advances, three key challenges continue to hinder the transition from quantitative tables to biological insights:

1. Limited feature resolution: Most downstream workflows remain primarily protein‐ or site‐centric, even though peptide‐level measurements can reveal regulatory information that is lost during protein‐group summarization. The protein‐level results are often disconnected from the underlying peptide, precursor, and phosphosite measurements needed to evaluate their quantitative support. As a result, researchers may have difficulty assessing whether biological interpretations are driven by robust experimental evidence or by summarization artifacts.
2. Missing biological context: Proteins function in different molecular and cellular contexts. However, the biological context of proteins is often neglected in statistical data analysis and visualization support. For instance, protein–protein interaction (PPI) and co‐expression networks are typically presented without subcellular localization annotations (McCaskie et al., 2025), making it difficult to evaluate whether the proteins occupy the same cellular compartment.
3. Tool fragmentation: Researchers often need to learn multiple tools for preprocessing, statistical testing, pathway enrichment, network analysis, and phosphoproteomics interpretation, each demanding its own file format, re‐annotation steps, and manual data transfer. Learning to use different tools or a programming language presents a significant barrier to researchers in the field.

Rather than introducing a new statistical framework, ProteoAnalyst was developed by integrating several well‐established methods with evidence tracing and visual analytics around two principles. First, each result should carry its biological context, including subcellular context (where in the cell a protein acts), mechanistic context (which pathways and kinases are involved), and regulatory context (which isoforms or modification states are changing). Second, every result should remain traceable to the evidence beneath it, so that any summary can be followed down to the individual proteins, peptides, precursors, phosphosites, and sample‐level measurements that support it. ProteoAnalyst applies both principles across the three levels at which proteomics data are interpreted, namely feature‐level, pathway‐level, and network‐level, pairing interpretation at each level with this context and one‐click drill‐down to the underlying measurements. The platform takes common upstream output tables through data processing, statistical analysis, and interactive visualization, and integrates statistical, functional, network, peptide‐level, phosphoproteomics, and biomarker analyses within a single environment, reducing the file conversion and re‐annotation that fragment current workflows. These workflows were evaluated through comprehensive benchmark case studies. The sections below describe how each level delivers context and traceability in practice, followed by four case studies. An overview of the ProteoAnalyst workflow is shown in Figure 1.

**FIGURE 1:**
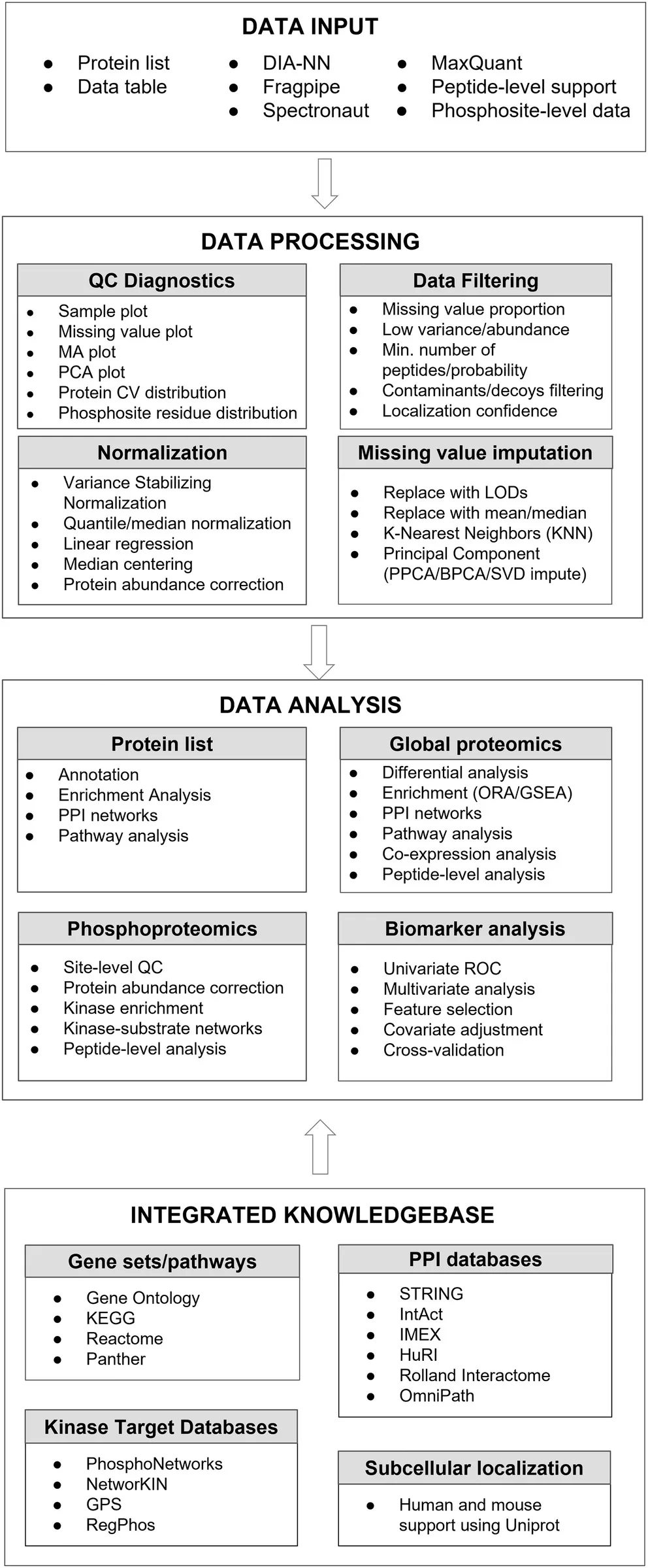
Overview of ProteoAnalyst's workflow.

## 2 MATERIALS AND METHODS

### 2.1 Platform overview

ProteoAnalyst follows the implementation of our widely used MetaboAnalyst platform (Pang et al., 2024). Briefly, the server‐side application is built using a Java/Jakarta EE framework. Data processing and statistical analysis are performed through an R package (https://github.com/xia-lab/ProteoAnalystR). The open‐source R package contains the complete analytical backend, and the release used for the analyses reported here is archived with the reproducibility bundle on Zenodo. The Java/Jakarta EE web application that provides the user interface and server layer is offered as a hosted service at www.proteoanalyst.ca; all statistical results reported here can be reproduced from the R package alone. To facilitate transparency and reproducibility, every analysis run records its underlying R command history together with a consolidated file (*analysis_summary.txt*) documenting its processing and statistical parameters. The platform includes step‐by‐step tutorials, embedded example datasets, frequently asked questions (FAQs), and a community forum. Example datasets are provided for global proteomics, phosphoproteomics, network analysis, and biomarker workflows, allowing users to explore the platform before uploading their own data. The public server accepts uploads of up to 150 MB per file. Users are encouraged to use the ProteoAnalystR package for large data and batch processing.

### 2.2 Data input and processing

ProteoAnalyst is designed to support common workflows in label‐free quantitative proteomics experiments. Depending on the input data types and research objectives, users can choose one of the four dedicated analysis modules: Protein List, Global Proteomics, Phosphoproteomics, or Biomarker Analysis. Apart from the protein list input, quantitative proteomics data must undergo a rigorous, multi‐step processing pipeline: data upload, quality control (QC), filtering, normalization, and missing value imputation. If peptide‐level data are uploaded, an additional peptide summarization step will be performed. In addition to generic protein abundance tables, ProteoAnalyst accepts the output files from common software tools including MaxQuant (*proteinGroups.txt* or *peptides.txt*), FragPipe (*combined_protein.tsv* or *combined_peptide.tsv*), DIA‐NN (*pg_matrix.tsv* or *report.tsv*), and Spectronaut (standard protein or peptide report files). In addition, a metadata file detailing group definitions and experimental design should also be uploaded. Users are encouraged to consult the built‐in example datasets for more details.

Data processing consists of the following key steps: initial data inspection, filtering, normalization, missing value imputation, and peptide summarization, when applicable. Feature filtering improves statistical power and enhances the robustness of differential analysis by eliminating features with excessive missing values, low variance, or insufficient annotation (Bourgon et al., 2010; Hackstadt & Hess, 2009). ProteoAnalyst provides diagnostic visualizations including abundance distribution boxplots, sample‐to‐sample distance dendrograms, principal component analysis (PCA) plots, and minus‐average (MA) plots. These plots allow users to assess data quality and evaluate the effects of normalization and missing value handling prior to downstream statistical analysis.

For identifier mapping and duplicate resolution, the feature IDs are mapped to the selected annotation type for lookup and enrichment while the original quantitative row labels are preserved. Specifically, UniProt isoform suffixes (*−n*) and phosphosite suffixes (*_Residue_Position*) are stripped solely for gene‐level lookup and enrichment, leaving the quantitative matrix intact. When multiple input rows resolve to the same analysis identifier, their values are aggregated sample‐wise by the mean. A detailed file documenting the ID annotation process can be exported. For every feature, it records the input identifier, the selected annotation identifier, its mapping status (mapped, unmapped, input‐accession‐preserved, or not‐attempted), whether it was retained in the quantitative matrix, the resulting analysis identifier, the size of its duplicate group, and the aggregation rule applied.

ProteoAnalyst supports sample‐level normalization to adjust technical variation such as sample loading, dilution, or global intensity shifts, followed by optional data transformation or feature‐level normalization. Available approaches for sample normalization include sample‐specific scaling factors, constant‐sum normalization, median normalization, and probabilistic quotient normalization (PQN) (Dieterle et al., 2006). Data transformation options include log2 transformation, log2 transformation with median centering (default), quantile normalization (Bolstad et al., 2003) and variance stabilizing normalization (VSN) (Huber et al., 2002). The choice of method should be guided by the expected structure of technical variation and evaluated using diagnostic plots before and after normalization. Further guidance on appropriate use cases, diagnostic cues, and cautions for each data transformation method is provided in Table S2.

ProteoAnalyst separates missing‐not‐at‐random (MNAR) and missing‐at‐random (MAR) choices. The default is MNAR limit‐of‐detection replacement: a missing value is replaced by one fifth of that feature's minimum positive observed intensity. Alternative left‐censored methods include MinDet, MinProb, and quantile‐regression imputation of left‐censored data (QRILC) (Peng et al., 2024). For data treated as MAR, the available established methods include feature mean or median replacement, K‐nearest neighbors (KNN), and Bayesian PCA (Method 1 in Appendix S1) (Lazar et al., 2016). For peptide‐level uploads, normalized peptides are summarized first, and the selected imputation method is then applied to the summarized protein matrix. Users should first examine whether missingness is intensity‐dependent, condition‐specific, or broadly distributed across samples. Left‐censored approaches are most appropriate when missing values are concentrated among low‐abundance features, whereas MAR‐oriented approaches are better reserved for cases where missingness is less tied to signal intensity or biological group. Features with excessive missingness should be filtered before imputation, and QC and differential results should be compared before and after imputation.

For peptide‐level data, normalized log2 peptide measurements are aggregated into protein‐level abundance estimates. The default is Tukey's median polish (TMP). Other options include the mean or median of log2 intensities, the log2 of summed linear intensities (Goeminne et al., 2015), as well as the Top‐N estimator (Silva et al., 2006) (Method 2 in Appendix S1). Because many peptides are shared across protein isoforms, protein‐level summarization can mask isoform‐specific evidence. ProteoAnalyst preserves peptide‐level traceability, including precursor identifiers and protein/transcript accession mappings when available, enabling users to inspect whether observed abundance changes are supported by the underlying peptides.

### 2.3 Data analysis and visualization

The following sections describe how ProteoAnalyst delivers context and traceability at each of the three levels of interpretation. (i) Feature level: differential expression testing, interactive exploration of significant features, and peptide‐level analyses that resolve changes lost to protein‐group summarization. (ii) Pathway level: over‐representation and gene set enrichment analyses that place these changes in biological context and project them onto interactive pathway maps. (iii) Network level: protein–protein interaction and co‐expression networks annotated with subcellular localization, situating the same features in their cellular context. Every level is coupled to interactive visualization, with most views supporting one‐click drill‐down to the proteins, peptides, precursors, phosphosites, and sample‐level measurements. Two application‐oriented modules, phosphoproteomics and biomarker candidate assessment, draw on all three levels and are described thereafter.

### 2.4 Feature‐level analysis

#### 2.4.1 Differential expression analysis

ProteoAnalyst implements a flexible interface to accommodate the common experimental designs based on limma (Ritchie et al., 2015), DEqMS (Zhu et al., 2020) or msqrob2 (Goeminne et al., 2020). limma fits a linear model per protein and shrinks each protein's residual variance toward a pooled estimate. DEqMS extends this by modeling the dependence of residual variance on the number of peptides or peptide‐spectrum matches (PSMs) quantifying each protein to yield count‐adjusted moderated statistics; if peptide/PSM counts are unavailable, the analysis falls back to the standard limma result (Method 3 in Appendix S1). Finally, msqrob2 instead models feature‐level intensities directly using robust regression. For data‐dependent acquisition (DDA) and other inputs with peptide/PSM counts, the default is DEqMS. For precursor‐level DIA inputs (DIA‐NN and Spectronaut), the default is msqrob2, which showed the most consistent performance among the integrated methods across our DIA spike‐in benchmarks (see Benchmarking analytical performance below); DEqMS and limma remain available and fully supported for DIA. The results are presented in various forms of interactive visualization coupled with functional analysis. Here we describe three common, yet complementary approaches.

#### 2.4.2 Exploring significant features

ProteoAnalyst provides an interactive volcano plot integrating statistical visualization with functional interpretation. The volcano plot displays log2 fold‐change (*x*‐axis) versus −log10(*p*‐value) (*y*‐axis), enabling rapid identification of features that are statistically significant with large effect size. Users can click on any data point to view a detailed boxplot showing expression distributions. For peptide‐level proteomics data, clicking on a protein reveals both an overview box plot and a detailed table of all constituent peptides with their individual fold‐changes and significance values, enabling investigation of peptide‐to‐peptide variability and identification of specific modified peptides driving protein‐level changes. Overall, the viewer allows users to select data points and perform enrichment analysis (Figure 2a). Alternatively, users can use a compartment‐organized Manhattan plot, in which features are grouped by subcellular localization rather than chromosomal position. For multi‐group experimental designs, users can use an UpSet diagram (Lex et al., 2014) to explore the overlap and uniqueness of significant features across multiple pairwise comparisons (Figure 2b). The interactive UpSet viewer supports three display modes (intersection, distinct, and union). Users can click on any set to perform enrichment analysis.

**FIGURE 2:**
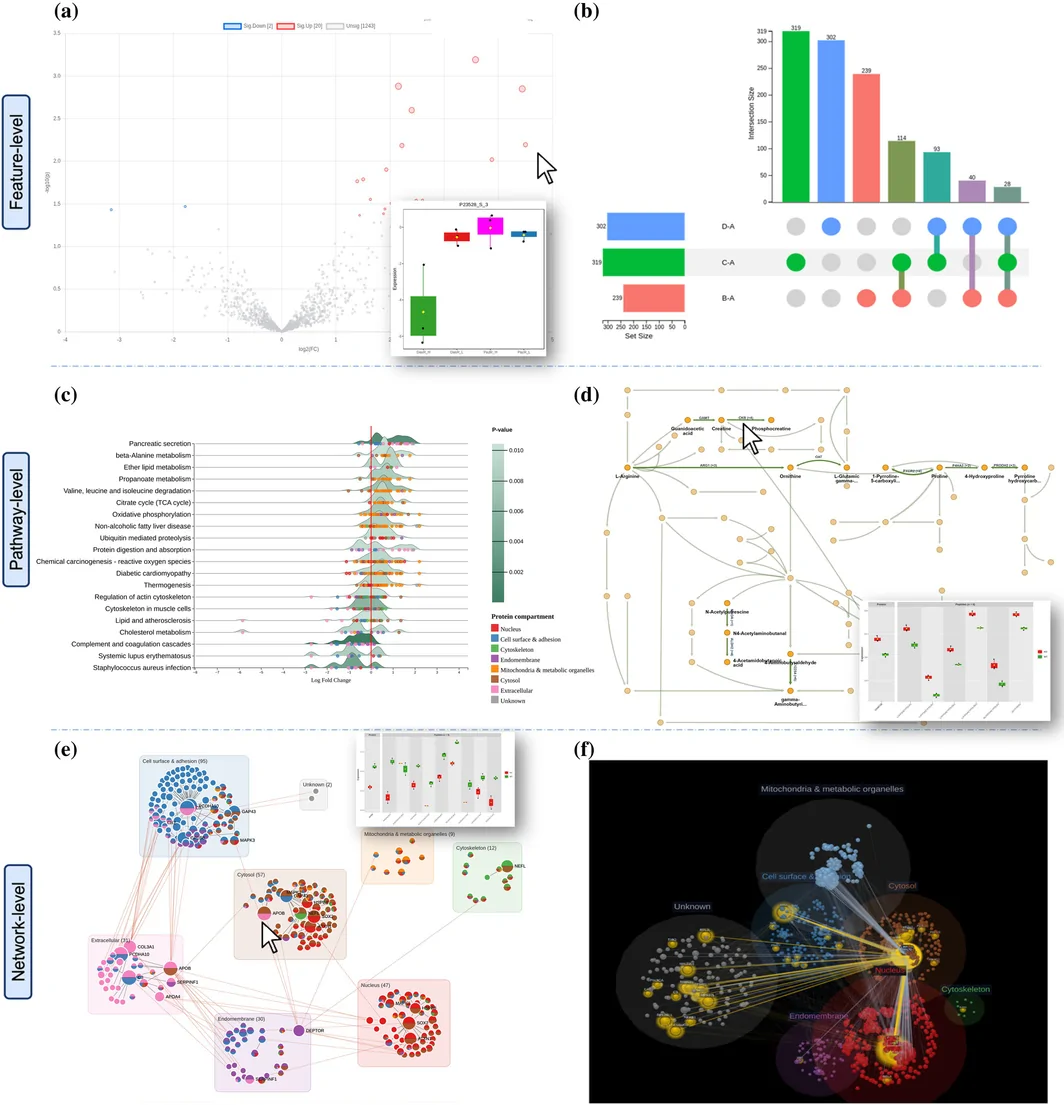
Visual analytics at feature, pathway and network‐level. All panels show protein‐level results from example datasets. One‐click drill‐down links any visual summary to the underlying proteins, peptides or phosphosites. (a) Volcano plot of proteins tested for differential abundance (significantly down‐ (blue) and up‐regulated (red) proteins are highlighted (FDR < 0.05 and absolute log2 fold change >1). Selecting a point opens a boxplot of that protein's abundance across samples. (b) UpSet diagram of the overlap and uniqueness of significant protein sets across pairwise contrasts. Bar height gives intersection size; selecting an intersection lists the constituent proteins and their enriched terms. (c) GSEA results as a ridgeline plot of enriched gene‐set terms, ranked by average fold change. Data points are the individual proteins contributing to each term, colored by their subcellular compartment. (d) KEGG pathway map of “Arginine and proline metabolism” with significant proteins projected onto pathway edges. Selecting an edge (enzyme) opens the abundance profile of the corresponding protein and its peptides (if available). (e) Two‐dimensional first‐order protein–protein interaction network in a subcellular‐compartment layout. Nodes are proteins; edges are physical interactions from STRING and curated interaction databases (IntAct, InnateDB, HuRI, and OmniPath). Selecting a node opens the abundance profiles of that protein and its peptides (if available). (f) Three‐dimensional view of the same network. Nodes matching an enriched gene set are highlighted in yellow.

#### 2.4.3 Isoform‐usage analysis

Peptide‐supported differential isoform‐usage analysis evaluates condition‐associated changes in relative isoform usage using proteotypic peptides and requires datasets searched against isoform‐aware databases, such as a UniProt canonical‐plus‐isoform database or OpenProt headers (Leblanc et al., 2024). Isoform‐level inference requires peptides that map uniquely to one isoform since digestion removes intact isoform context and renders shared peptides ambiguous (Li & Radivojac, 2012; Schaffer et al., 2019). ProteoAnalyst retains precursors annotated as proteotypic and links them to their protein accession, gene, and transcript tags when available. The function queries whether the relative usage of an isoform shifts between conditions using proteotypic peptides that map uniquely to each isoform. The approach has adapted the isoform‐switching concept from transcript‐level analysis to continuous label‐free peptide intensities (Schaffer et al., 2019). A switch requires both tests to pass FDR < 0.05, at least two distinct proteotypic peptide sequences per isoform (charge states and modified forms of one sequence count once). Both the numbers of distinct peptides and the number of precursors supporting each isoform are reported, and no accompanying change in overall protein abundance (Method 4 in Appendix S1).

#### 2.4.4 Relative modification‐fraction analysis

Relative modification‐fraction analysis is currently designed for DIA‐NN precursor‐level data. It identifies modified and unmodified forms of the same peptide co‐detected in the same sample. Two tests are performed. First, for each matched pair, the platform calculates the modified signal relative to the total peptide signal (the relative modification fraction) and tests whether it differs between conditions. Second, a separate test on total peptide abundance determines whether the apparent change can be explained by an overall change in peptide abundance. This is conceptually related to protein abundance correction in enriched phosphoproteomics (Wu et al., 2011), but it operates directly on paired peptide forms observed in unenriched global proteomics data. Results across all detected modified/unmodified peptide pairs are displayed in a Manhattan plot with sites grouped by their subcellular compartments. Because modified and unmodified forms of a peptide can differ in ionization efficiency and no analyte‐specific calibration is applied, this fraction is a relative measure for comparing conditions; it should not be interpreted as absolute site occupancy or modification stoichiometry.

### 2.5 Pathway‐level analysis

In addition to over‐representation analysis, ProteoAnalyst supports Gene Set Enrichment Analysis (GSEA) as a cutoff‐free option for global proteomics and phosphoproteomics. GSEA evaluates coordinated pathway‐level abundance patterns without requiring a significance cutoff. ProteoAnalyst uses the *fgsea* R package on all mapped measured features ranked by either fold change or *t*‐statistics; after identifier mapping, duplicate gene identifiers retain the entry with the largest absolute statistic (Korotkevich et al., 2016; Subramanian et al., 2005). Users can perform GSEA coupled with interactive visualization using ridgeline diagrams (Figure 2c) or clustering heatmaps. Beyond these enrichment summaries, ProteoAnalyst can project protein abundance changes onto an interactive pathway map (Figure 2d), where users can select an enzyme to drill down to the expression of its underlying proteins, linking pathway‐level patterns to the underlying protein abundance measurements. Enrichment analyses are based on Gene Ontology (Gene Ontology Consortium, 2026), KEGG (Kanehisa et al., 2025), Reactome (Ragueneau et al., 2026), or PANTHER (Thomas et al., 2022). The background universe is the measured proteome or phosphoproteome due to limited coverage for most current proteomics platforms (Appendix S1). Database versions and download dates are listed in Table S3.

### 2.6 Network‐level analysis

#### 2.6.1 Interactive PPI networks with subcellular compartment annotation

ProteoAnalyst supports protein–protein interaction (PPI) network creation and visualization. It currently offers nine network layout algorithms to help explore the results. For instance, users can choose “Compartment layout”, and proteins sharing the same cellular compartments will be positioned within the same rectangular region based on their UniProt subcellular localization annotations (UniProt Consortium, 2025). Each region is color coded and displays the number of proteins contained (Figure 2e). A multi‐localized protein is drawn once in every compartment it has been assigned to. The bottom half of the node is divided into pie wedges colored by the other compartments the protein occupies. The full compartment membership of each protein is preserved in the node attributes and exported in the node table. Different node coloring can be applied to enhance visualization and interpretation. Users can manually highlight a compartment or an area of interest and perform functional enrichment analysis. Users can also double‐click on a protein node to display its associated peptides (when available). In the “Current selection” panel on the bottom right corner, users can click on “View boxplot” to inspect the intensity distribution of the protein or peptide. Users can also view the interactive network in 3D space (Figure 2f). Physical interactions are drawn from STRING (default combined‐score threshold ≥900, high confidence; user‐adjustable) (Szklarczyk et al., 2025) together with IntAct (Kerrien et al., 2012), InnateDB (IMEx‐curated interactome) (Breuer et al., 2013), HuRI (Luck et al., 2020) and OmniPath (Türei et al., 2021). For trimming large networks, the prize‐collecting Steiner forest module uses the PCSF formulation (Akhmedov et al., 2017).

#### 2.6.2 Co‐expression network analysis and integration with PPI

ProteoAnalyst supports protein co‐expression network analysis using CEMiTool, built on the widely used weighted gene co‐expression network analysis (WGCNA) framework (Langfelder & Horvath, 2008; Russo et al., 2018). To limit the computational cost of network construction, datasets containing more than 5000 features were restricted to the top 5000 features with the largest interquartile range. The soft‐thresholding power was selected automatically using CEMiTool's scale‐free‐topology procedure. The correlation‐based module detection is unstable in small cohorts and is only recommended with at least 20 samples.

Multiple diagnostic plots and visualizations are also provided to facilitate interpretation of co‐expression patterns. For instance, the module dendrogram displays hierarchical clustering of samples based on module eigengene expression profiles, while the module‐trait heatmap shows correlation strengths between module eigengenes and sample phenotypes. Before proceeding to the network viewer, users can optionally add PPI information onto the co‐expression network. Edges that overlap with known PPI interactions are highlighted in orange, providing biological support of co‐expression relationships. Similar to the PPI network view, users can double click a node and use the “View boxplot” under the “Current selection” panel to inspect the intensity distribution of the protein and underlying peptides. By integrating PPI networks and co‐expression networks together with cellular compartments, ProteoAnalyst enables researchers to uncover biological insights from complementary perspectives: curated physical interactions versus observed expression correlations in their specific subcellular context.

### 2.7 Phosphoproteomics workflow

This module is designed to bridge site‐level quantitative changes with pathway‐ and network‐level mechanisms. It accepts phosphosite‐centric quantitative inputs alongside sample metadata. The workflow mirrors the global proteomics module including QC, normalization, differential analysis, and an additional step to map phosphosite identifiers to parent proteins. This step enables downstream enrichment while maintaining traceability back to the original peptide‐level evidence.

#### 2.7.1 Data filtering and normalization

To address the technical challenges of phosphoproteomics (e.g., enrichment bias and sparse data patterns), this module introduces specialized preprocessing steps including localization filtering (i.e., retaining only confidently localized phosphorylation sites), and protein abundance correction (i.e., isolating the relative phosphosite change from confounding shifts in baseline protein expression).

#### 2.7.2 Protein‐adjusted phosphosite change

For phosphoproteomics datasets with matched total‐proteome reference data, ProteoAnalyst estimates the protein‐adjusted relative phosphorylation change by evaluating phosphosite intensity relative to parent protein abundance, providing a protein‐abundance‐adjusted measure of site‐level phosphorylation change (Method 6 in Appendix S1). This phosphosite/protein‐reference workflow is not restricted to DIA‐NN: MaxQuant phosphoproteomics and DIA‐NN phosphoproteomics inputs are supported as long as the corresponding protein reference can be loaded, such as MaxQuant proteinGroups.txt or DIA‐NN pg_matrix.tsv from the matched total‐proteome experiment. In this setting, phosphosite intensity is evaluated relative to the corresponding parent protein abundance, producing a log2 phosphosite/protein abundance ratio that helps separate site‐level phosphorylation changes from substrate protein abundance changes. This workflow is conceptually related to protein abundance correction and MSstatsPTM‐style analyses for enriched phosphoproteomics data (Kohler, Tsai, et al., 2023; Maier et al., 2025), whereas the paired‐peptide workflow uses co‐detected modified and unmodified precursor forms directly. Together, these approaches help identify condition‐dependent changes in relative modification levels while preserving traceability to the underlying peptides, phosphosites, parent proteins, and sample‐level measurements.

#### 2.7.3 Kinase enrichment analysis

The module tests annotated kinase substrate sets for enrichment among selected phosphosites and summarizes coordinated changes in substrate phosphorylation (Figure 3a). ProteoAnalyst currently integrates four local kinase–substrate annotation resources: PhosphoNetworks (Hu et al., 2014), NetworKIN (Linding et al., 2007), GPS (Group‐based Prediction System) (Chen et al., 2023), and RegPhos (Huang et al., 2014). To ensure stable performance, ProteoAnalyst does not execute external prediction tools or web services at runtime. These resources are used as local curated or predicted kinase–substrate annotation sets selected by the user. Results based on predicted annotations should therefore be treated as exploratory hypotheses.

**FIGURE 3:**
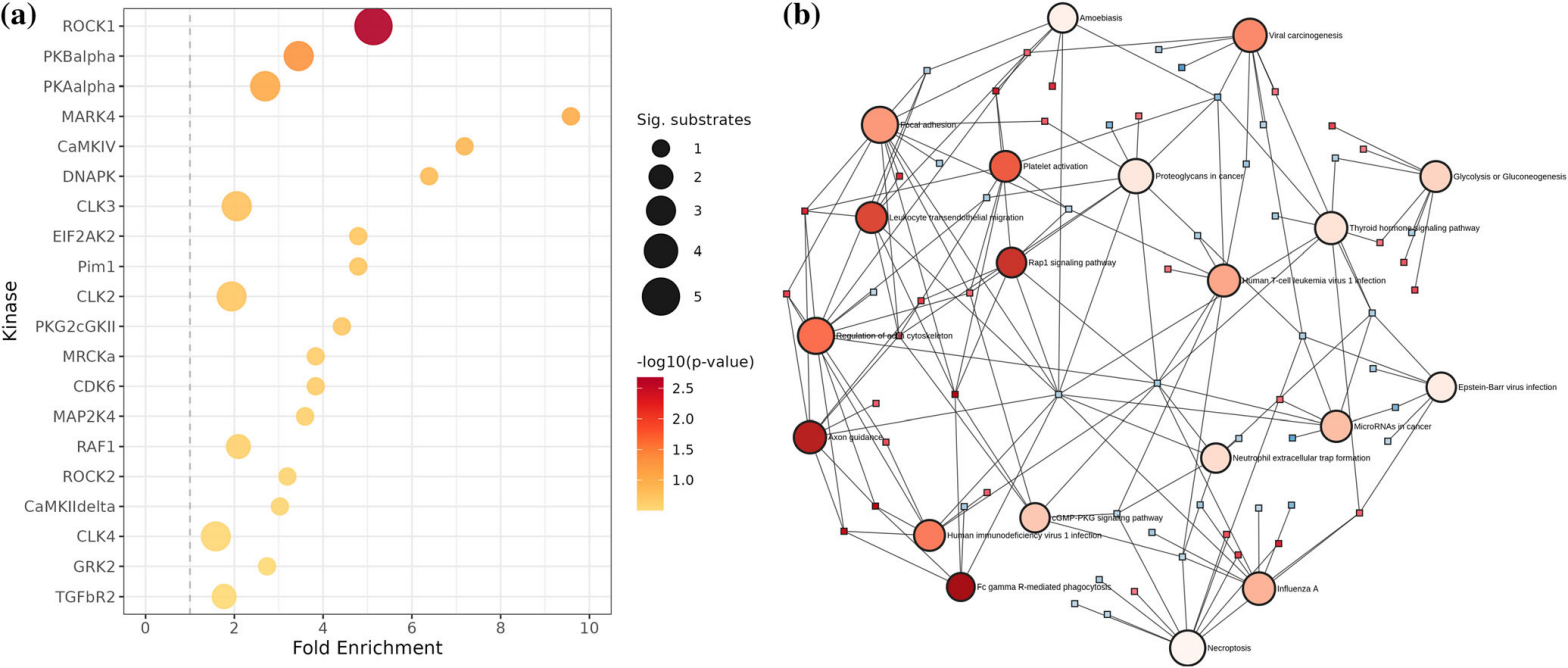
Kinase substrate‐set and KEGG pathway enrichment for the interaction contrast. (a) NetworKIN kinase substrate‐set over‐representation. Point size indicates the number of nominally altered substrates in the set and color the raw ORA *p*‐value (−log10 scale). No substrate set passed BH FDR < 0.05, so the kinase‐level results are exploratory. (b) KEGG gene‐set enrichment analysis of the nested interaction contrast: Protein‐adjusted site log2 fold changes are collapsed to one value per gene (largest absolute change) and the ranked gene list is tested against KEGG pathways. Node size indicates the number of pathway genes present in the ranking and color the FDR (more significant is in dark red).

For kinase, compartment, and motif enrichment, each result records the number of selected phosphosites, the selection criteria, as well as the analysis‐specific measured‐site universe and its size. Over‐representation uses a one‐sided hypergeometric test, with raw p‐values adjusted across all tested terms by the Benjamini–Hochberg method. Kinase analysis additionally reports the signed KSEA *z*‐score (Casado et al., 2013). Overall, these analyses summarize substrate‐level phosphorylation patterns and should not be interpreted as direct measurements of kinase enzymatic state.

These annotated relationships can be explored through interactive kinase–substrate networks. Central kinase nodes are linked to their annotated substrate phosphosites to display statistical support for coordinated substrate hyper‐ or hypo‐phosphorylation. Each kinase node is colored by its log2 fold‐enrichment score. Double‐clicking a kinase node reveals its associated phosphosites (colored by log2 fold‐change via a blue‐to‐red gradient), while double‐clicking a phosphosite node displays its corresponding quantitative intensity boxplot. Additionally, users can toggle a bipartite view mode to expand all kinase nodes simultaneously, displaying the complete network. This view connects site‐level phosphorylation changes with kinase‐substrate annotations to facilitate hypothesis generation and targeted validation.

### 2.8 Biomarker candidate assessment

This module supports translational proteomics studies in which the primary objective is candidate biomarker identification and performance evaluation. Users can conduct classical univariate analysis to generate receiver operating characteristic (ROC) curves for any protein or peptide of interest and explore their sensitivity and specificity across different cutoffs. For multivariate biomarker discovery, the platform couples different feature selection algorithms with supervised machine learning models, including logistic regression, elastic net (Friedman et al., 2010), random forests, support vector machines (SVMs), and partial least squares‐discriminant analysis (PLS‐DA). Model performance is evaluated by stratified Monte Carlo cross‐validation with balanced subsampling (Xia et al., 2013), making it effective for the limited sample sizes and imbalanced groups often found in clinical datasets. Users can inspect top‐performing models including cross‐validated Area Under the Curve (AUC) metrics with 95% confidence intervals and biomarker stability based on the feature selection frequency across folds.

Clinical and experimental cohorts often contain additional phenotypic metadata (e.g., age, sex, BMI, or location) that can improve predictive performance. ProteoAnalyst allows users to include these variables directly as features alongside the molecular data. These covariate features undergo the same fold‐contained feature selection, model training, and permutation testing as the protein markers. Because no global covariate adjustment is applied to the raw molecular data, all preprocessing remains strictly confined within individual training folds, preventing data leakage.

## 3 RESULTS

### 3.1 Benchmarking analytical performance

Because ProteoAnalyst integrates established statistical methods rather than introducing a new statistical framework, we evaluated how its workflows compare with established implementations in terms of differential expression analysis. Five controlled benchmarks, summarized in Table S4, spanned the platform's acquisition modes: two DDA/MaxQuant spike‐ins (iPRG‐2015, Table S5a; and the MaxLFQ two‐species proteome mixture, PXD000279, Table S5b; Text 1 in Appendix S1), two DIA/Spectronaut mixtures (LFQbench HYE124, Table S6a; and a UPS1‐in‐*E. coli* spike‐in, MSV000087597, Table S6b; Text 2 in Appendix S1), and a two‐species phosphoproteomics spike‐in (PXD007145). Dataset descriptions, ground‐truth definitions, and full result tables are given in Appendix S1. Performance was assessed using fold‐change accuracy, sensitivity, specificity, empirical FDR (the observed fraction of false discoveries among features called significant at the nominal 5% threshold), ROC AUC, missing‐value robustness (Table S7 and Text 3 in Appendix S1), and effect‐estimate concordance. Because the asymmetric spike‐in design of PXD000279 induces a composition shift that species‐agnostic normalization redistributes into the unchanged human background, this benchmark was scored with metrics robust to such shifts (species ROC AUC and fold‐change separation against the design ratio), following the evaluation approach of the dataset's originating study (Cox et al., 2014).

ProteoAnalyst's DDA and DIA defaults were evaluated separately from comparison runs that used reference preprocessing. As independent external references, MSstats and MSstatsPTM (Kohler, Staniak, et al., 2023; Kohler, Tsai, et al., 2023) were run on the same inputs (Tables S5a, S8 and S9; Text 4 and 5 in Appendix S1). Because ProteoAnalyst embeds several differential expression methods, we additionally benchmarked those methods against one another (limma, DEqMS, and msqrob2) under identical data processing to assess performances. No single workflow performed best under all conditions: on the LFQbench HYE124 mixture, for example, the MSstats reference outperformed ProteoAnalyst's DIA default on every scored metric, including empirical FDR (0.063 vs. 0.100) and ROC AUC (0.965 vs. 0.937; Table S6a). The benchmarks are therefore intended to show that ProteoAnalyst provides established methods that its default settings perform competitively against other widely used platforms, with an intuitive user interface and visualization to allow further customization based on data and analysis objectives. Runtime and scalability of the differential‐expression step were profiled against protein count on the iPRG‐2015 matrix (Table S10 and Text in Appendix S1). The benchmarks were executed through the same R backend used by the web application, with fixed truth definitions and common scoring rules. Public accessions, analysis parameters, session information, and scoring scripts are provided in the accompanying reproducibility bundle (Zenodo, https://doi.org/10.5281/zenodo.22260116).

### 3.2 Case studies

We illustrate ProteoAnalyst usage with four datasets, each chosen to demonstrate a different level of the platform. The first, a previously unpublished study of palmitate‐treated human and stem cell‐derived islets, illustrates the core statistical and functional workflow, recovering previously reported biology. The second, a global proteomics study of FB231‐induced mitophagy, shows how enrichment and protein interaction network analysis can surface unexpected biological context. The third, a phosphoproteomics study of tyrosine kinase inhibitor (TKI) resistance, demonstrates the phosphoproteomics workflow from protein abundance correction through kinase enrichment and kinase‐substrate networks. The fourth, a DIA dataset of knockout and matched wildtype cell lysates, demonstrates peptide‐level isoform‐usage and PTM relative modification‐fraction screening. Each case‐study analysis is packaged as a self‐contained reproducibility bundle and deposited on Zenodo (DOI: https://doi.org/10.5281/zenodo.22260116). Every bundle contains the input data, the analysis configuration, rerun script, and the expected outputs. The deposit also includes the ProteoAnalystR source package and a lock file pinning the full R environment, so any case study can be re‐executed end to end with a single command. Values quoted below are identified as nominal or FDR‐adjusted; findings supported only by nominal significance are treated as exploratory.

#### 3.2.1 Case study 1: Effects of palmitate incubation on human islets and stem cell‐derived islets

The statistical and functional workflow of ProteoAnalyst was first assessed using a DIA‐PASEF proteomics dataset from human islets (five donors; Table S14) and stem cell‐derived islets (SC‐islets) treated for 24 h with 0, 0.25, 0.5 or 0.75 mM BSA‐complexed palmitate. Data were normalized by log2 transformation and median normalization, with missing values imputed using the default limit‐of‐detection method. In line with earlier reports, the proteomes of human islets clustered distinctly from SC‐islets in a PCA (Kolic et al., 2024), while palmitate incubation produced only modest proteome changes. Notably, the majority of the top‐ranked proteins by *p*‐value in human islets, and a substantial fraction in SC‐islets, matched our earlier islet proteomics study (Kolic et al., 2024), indicating that ProteoAnalyst recovers known biology despite differences in treatment parameters and analysis methods. Differential analysis identified no significant features in SC‐islets following palmitate incubation, whereas five proteins changed in human islets (FDR < 0.05 at one or more doses vs 0 mM): FAR1, PLIN2 and ZCCHC3 increased, and SLC46A1 and SLC35E1 decreased. Overrepresentation analysis (ORA) highlighted “vitamin digestion and absorption”, “mineral absorption”, “antifolate resistance”, and “PPAR signaling”, and both mineral absorption and PPAR signaling have been reported in human islets similarly exposed to palmitate (Groebe et al., 2018; Sargsyan et al., 2018). Together, these results are consistent with published findings, providing a baseline of confidence for the more specialized analyses that follow.

#### 3.2.2 Case study 2: Global proteomics of FB231‐induced mitophagy

Here, we used ProteoAnalyst to analyze a global proteomics data obtained from HeLa cells treated with the mitophagy activator FB231, which stimulates Parkin via PINK1. Parkin is an E3 ubiquitin ligase implicated in mitochondrial quality control and Parkinson's disease, making the discovery of activators a desirable therapeutic strategy. Data were analyzed using FragPipe's LFQ‐MBR pipeline, under the default settings including MaxLFQ normalization (Cox et al., 2014) and imported into ProteoAnalyst at the peptide level for filtering, summarization, and differential analysis. Initial quality assessment in ProteoAnalyst flagged inconsistent clustering of the FB231 conditions in the PCA; replicate grouping improved after standard feature filtering and normalization. In the original study, the authors suggested that FB231 induces both mitophagy and the integrated stress response (ISR) by chelating iron (Rosencrans et al., 2025), similar to deferoxamine (Sekine et al., 2023). They notably observed perturbation in iron‐responsive proteins such as IREB2 (up‐regulated) and FECH (down‐regulated), which we also observed in our own analysis (exploratory; not significant after FDR correction). Consistent with the role of FB231 as a mitophagy activator, the subcellular compartment‐stratified significance plot organizes protein‐level differential significance by cellular location and highlights members of the mitochondrion organization gene set (Figure 4a), and the accompanying expression heatmap displays the relative intensity of these mitochondrion organization proteins across the DMSO and FB231 replicates (Figure 4b). We also identified many processes linked to cell division, mitosis, and spindle organization. PPI network analysis with the STRING interactome showed substantial involvement of nuclear and spindle proteins, consistent with changes in cell cycle regulation. Whether this is induced by iron depletion is a consequence of mitophagy or ISR activation is an interesting follow‐up. This analysis shows how ProteoAnalyst can surface candidate processes for experimental follow‐up.

**FIGURE 4:**
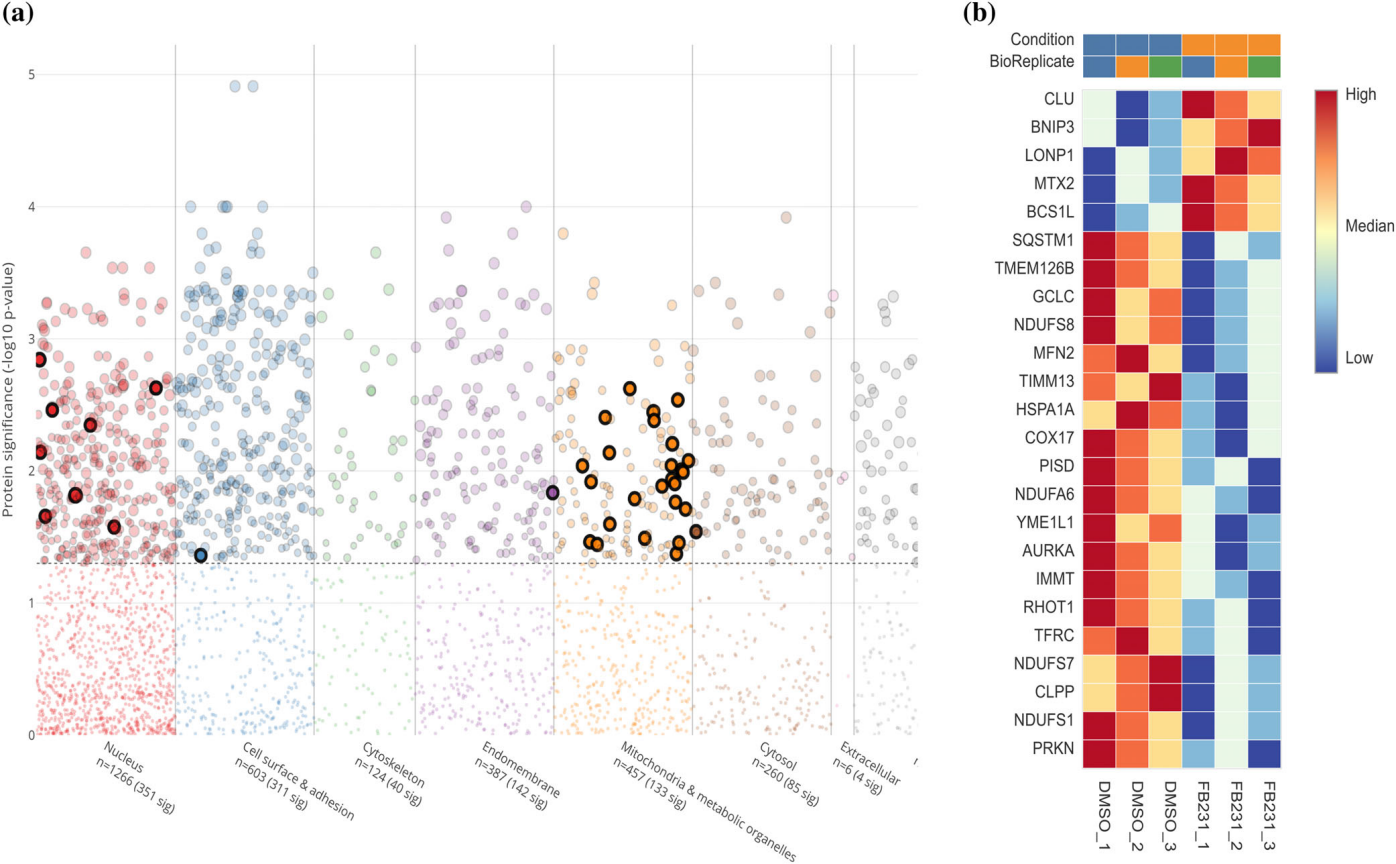
Subcellular compartment‐stratified significance plot and expression heatmap of the mitochondrion organization gene set. (a) Manhattan‐style plot depicting protein significance (−log10 *p*‐value) organized by subcellular compartments along the *x*‐axis. Each circle represents a protein, colored by compartment. Proteins belonging to the mitochondrion organization gene ontology term are highlighted with outlined circles. (b) Heatmap showing the relative intensity of mitochondrion organization gene set members across samples. Columns represent individual biological replicates grouped by condition. Rows represent individual proteins. Color scale reflects normalized abundance levels from low (blue) to high (red).

#### 3.2.3 Case study 3: Phosphoproteomics analysis of tyrosine kinase inhibitor resistance

We used ProteoAnalyst to analyze data from a phosphoproteomics study of acquired resistance to pazopanib and dasatinib in A204 rhabdoid tumor cells (Vyse et al., 2018). The experimental design is a two‐channel (duplex) SILAC phosphoproteomics setup in which resistant cells (dasatinib‐resistant or pazopanib‐resistant) were heavy‐labeled (R10K8) and mixed 1:1 with light‐labeled (R0K0) parental A204 cells prior to digestion and phosphopeptide enrichment with each resistant line profiled in a separate set of three biological replicate runs against the shared parental background. Note that although ProteoAnalyst doesn't perform isotope‐channel quantification, it does accept the processed ratio matrix that a SILAC search engine produces. The original study found that <10% of the quantified phosphoproteome was altered upon acquisition of drug resistance, with pazopanib‐resistant cells displaying elevated phosphorylation in cytoskeletal regulatory pathways while dasatinib‐resistant cells showed upregulation of insulin receptor/IGF‐1R signaling components, indicating distinct resistance mechanisms.

Data were uploaded to ProteoAnalyst in MaxQuant Phospho(STY)Sites format, normalized using global proteome abundance correction, and missing values were handled by hybrid (MAR/MNAR) imputation. A limma contrast was then used to compare the resistant‐versus‐parental SILAC response between the two drug‐resistance contexts, effectively estimating (pazopanib‐resistant/parental) − (dasatinib‐resistant/parental) on the log2 H/L scale. Phosphosites significant for this contrast therefore represent divergent phosphorylation responses between the two resistance mechanisms rather than sites changing in either resistant line alone. No sites passed an FDR threshold of 0.05; the contrast identified 22 nominally altered phosphosites (raw *p* < 0.05, of 1265 sites tested) exhibiting different phosphorylation dynamics between the two TKI resistance mechanisms, with the largest changes at actin‐regulatory sites: the inhibitory Ser3 of cofilin‐1 (P23528_S_3, log2FC = −4.5) and cofilin‐2 (Q9Y281_S_3), the cofilin‐family member destrin (P60981_S_3), and VASP S239 (P50552_S_239). ORA using gene ontology biological processes revealed regulation of actin filament length as the top enriched term (adjusted *p* = 6.2 × 10^−6^), with actin filament‐based process also enriched (adjusted *p* = 0.004); the contributing proteins are highlighted on the volcano plot and the intensity distribution of the cofilin‐1 Ser3 phosphosite is shown in Figure 2a. The exported site table retains the feature‐level fold changes and raw and BH‐adjusted *p*‐values. Because no sites pass FDR < 0.05, the analysis results are interpreted as exploratory.

NetworKIN kinase–substrate analysis ranked annotated substrate sets among the nominally altered phosphosites (Figure 3a); no substrate set passed BH FDR < 0.05, so the kinase‐level results are likewise exploratory. The top‐ranked set was ROCK1 (five of its 56 annotated substrates among the nominal sites; raw *p* = 0.002, FDR = 0.29), consistent with the actin‐regulatory signature of the site‐level results, since ROCK signaling converges on cofilin phosphorylation through LIM kinases. Substrate sets for stress‐linked kinases were not significantly enriched in this contrast: the IKKβ set contained no nominally altered substrates, despite the reported capacity of mitochondrial stress to engage IKK activation (Harding et al., 2023), and CaMKII‐family sets were likewise not enriched, although CaMKII can be directly activated by reactive oxygen species through oxidation of a conserved methionine residue (Erickson et al., 2008).

GSEA using KEGG pathways identified Fc gamma R‐mediated phagosome formation and Pertussis as the top enriched terms (adjusted *p* = 0.02 each), followed by axon guidance (nominal *p* = 0.004; adjusted *p* = 0.19) and further nominally enriched actin‐ and adhesion‐related terms (Rap1 signaling, leukocyte transendothelial migration, platelet activation, and regulation of the actin cytoskeleton) (Figure 3b). These results complement the ORA findings: the enriched terms share a leading edge of actin regulators (the cofilins CFL1/CFL2 and VASP), and actin remodeling is mechanistically linked to both phagosome formation and axon guidance (Schneider et al., 2023). Overall, this case study illustrates ProteoAnalyst's phosphoproteomics workflow from statistical analysis to exploratory biological interpretation.

#### 3.2.4 Case study 4: Peptide‐level regulatory analysis using a cell‐lysate dataset

To detect isoform‐level changes, whole cell lysates from a Parkin knockout and matched wildtype line profiled by DIA were searched against the OpenProt database (Leblanc et al., 2024), which retains reference proteins, canonical isoforms, and alternative translation products annotated with transcript (TA) and protein accession (PA) identifiers, extending coverage beyond the standard UniProt reference proteome. The DIA‐NN precursor‐level output was filtered to include only proteotypic peptides so that isoform‐level quantification rests on unambiguous evidence.

Isoform‐usage analysis was applied to screen for condition‐dependent shifts in relative isoform usage across proteins with proteotypic peptide evidence. Under the default evidence criteria, five gene‐isoform pairs were testable in this dataset, and in every pair the minor isoform was supported by exactly two distinct peptides. No pair showed statistically significant differential isoform usage after FDR correction (minimum ratio FDR = 0.058); the strongest candidates (RBBP4, PHLDB2, and TNRC6B; nominal *p* = 0.017–0.046) are reported as exploratory shifts in isoform balance rather than complete isoform switches. A null result at stringent evidence thresholds is expected for a three‐versus‐three comparison: alternative isoforms are rarely resolved at the peptide level in bottom‐up proteomics, where the great majority of genes present a single dominant isoform (Abascal et al., 2015; Ezkurdia et al., 2015), and the analysis is deliberately restricted to unambiguous dual‐isoform peptide evidence, with every candidate traceable to its supporting precursors for orthogonal follow‐up.

Relative modification‐fraction analysis estimates the fraction of a peptide's measured signal that carries a given modification. This distinguishes shifts in the modified fraction, where the modification rate changes independently of protein level, from cases where a modification simply tracks the abundance of its host protein. Relative modification fractions were computed across 2619 peptide‐modification pairs: 2581 methionine oxidation, 35 acetylation and 3 combined events, with between‐condition changes assessed by *limma* on logit‐transformed fractions and a separate *limma* test on total peptide abundance. Because methionine oxidation can also arise during sample preparation, chromatography and ionization, these changes are interpreted as exploratory. The compartment‐organized Manhattan plot displays the significance of modification‐fraction changes for all detected PTM peptide pairs organized by subcellular compartment, providing a proteome‐wide view of the distribution of modification changes across cellular locations. One site passed FDR correction: a methionine in the IQGAP1 peptide TLINAEDPPMVVVR showed a relative modification fraction that increased from 0.03 in WT to 0.35 in KO (FDR = 0.002) with no change in total peptide abundance (*p* = 0.16). The next‐ranked sites (GOLM2 and FASN peptides, FDR = 0.06) illustrate the case of confounded abundance: both coincide with significant changes in total peptide abundance. Beyond individual sites, the signal is broad: most oxidation events (93%; 2411 of 2581) showed a higher relative modification fraction in KO relative to WT, with a mean increase of approximately 0.12 among the 352 oxidation sites reaching nominal significance (*p* < 0.05). This uniform shift in direction also raises the possibility of systematic technical oxidation, for example from differences in sample handling, preparation or processing batch between the KO and WT samples, and the public dataset provides no independent evidence (such as blocking of unoxidized methionines) that would exclude it. Among the most affected proteins with known localization are several resident mitochondrial enzymes (Rath et al., 2021), NDUFS7, IDH2 and ECHS1, offered as illustrative examples.

The sites can also be viewed as a compartment‐organized Manhattan plot, in which features are grouped by subcellular localization rather than chromosomal position. The nominally changed oxidation sites are distributed across compartments, with mitochondria and metabolic organelles the largest annotated group (71 of 352 sites), followed by nuclear and endomembrane locations; no single compartment dominates, and compartment membership itself is not significantly enriched relative to all quantified oxidation sites. Functional enrichment, in contrast, does rise above background: ORA of the proteins carrying nominally changed oxidation sites, tested against the 3360‐protein measured proteome, yields 20 GO biological‐process terms at FDR < 0.05, led by aerobic respiration (FDR = 0.004), cellular respiration, and energy derivation by oxidation of organic compounds (FDR = 0.01). This pattern is compatible with an increased oxidative burden following Parkin loss. However, oxidation‐controlled sample preparation is necessary to rule out technical artifacts and confirm Parkin‐dependent biology.

## 4 DISCUSSION

### 4.1 Comparison with other tools

We compared ProteoAnalyst with several representiative platforms developed for quantitative proteomics analysis, including Perseus (Tyanova et al., 2016), MSstats (Kohler, Staniak, et al., 2023), the Monash Analyst Suite (Hsiao et al., 2024; Shah et al., 2019; Zhang et al., 2023), and ProteoArk (Nisar et al., 2025). These tools can be grouped into desktop environments, statistical frameworks, workflow‐specific web applications, and integrated web platforms. They address important and partially overlapping needs, but each leaves a different segment of the path from quantitative table to biological hypothesis unaddressed. A feature‐level summary of these capabilities across tools is provided in Table S1.

Perseus is a mature desktop environment tightly integrated with MaxQuant outputs, providing extensive support for matrix processing, imputation, statistical testing, clustering, enrichment, and plugin‐based extension. While these strengths have made it a highly influential platform with substantial documentation and training material, its focus is primarily at protein and matrix levels; it does not provide compartment‐aware network analysis, or web‐based navigation from a biological summaries back to the underlying peptide measurements.

MSstats is an R/Bioconductor framework for model‐based quantification across label‐free, targeted, DIA, and labeled workflows, with MSstatsPTM (Kohler, Tsai, et al., 2023) extending it to post‐translational modifications and MSstatsShiny (Kohler, Kaza, et al., 2023) adding a reproducible point‐and‐click interface. Its core strength lies in rigorous statistical modeling, including feature summarization, normalization, missing‐value handling, and differential testing across diverse acquisition and design types, whereas downstream functional, network, compartment, and biomarker interpretation are largely outside its scope.

The Monash Analyst Suite provides focused web applications for rapid, guided analysis of specific upstream output types: namely LFQ‐Analyst, DIA‐Analyst, TMT‐Analyst, Phospho‐Analyst, and FragPipe‐Analyst. A separate Pathway‐Analyst application supports functional enrichment, KEGG pathway diagrams, and STRING interaction networks. Although effective within their respective formats, these tools are modular and fragmented. In contrast to ProteoAnalyst, transitioning from quantitative results to biological pathway or network context requires exporting data between applications, limiting seamless, linked inspection of the underlying peptide‐level evidence (Table S1).

ProteoArk is an integrated web platform that processes standard outputs from Proteome Discoverer, MaxQuant, and MSFragger through quality control, normalization and batch correction, differential expression, and visualization, and it also supports transcriptomic, genomic, and metabolomic data. Although it is the closest in scope to an all‐in‐one platform, it does not currently offer native phosphoproteomics and PTM analysis, peptide‐level isoform analysis, or subcellular compartment network layouts.

ProteoAnalyst is designed to bridge these gaps within a single environment. Users can move seamlessly from data processing and differential analysis to enrichment, network exploration, and phosphoproteomics interpretation, and to peptide‐level isoform and relative modification‐fraction screening and biomarker evaluation without repeated file conversion or re‐annotation. Two features are worth highlighting. First, ProteoAnalyst provides biological context at every level, including interaction networks grouped by subcellular compartment and kinase‐substrate networks. Second, it preserves evidence traceability so that any pathway, network, kinase, biomarker, isoform, or modification‐fraction result can be followed back to the individual proteins, peptides, precursors, phosphosites, and sample‐level measurements that support it. Together, these capabilities allow the same dataset to be interrogated for what changed, where in the cell it occurs, how it connects to known regulatory mechanisms, and at what molecular resolution.

## 5 CONCLUSIONS

ProteoAnalyst addresses a recurring need in modern proteomics: converting quantitative output tables into interpretable biological evidence. Its design treats biological context and evidence traceability as guiding principles applied at every level. These principles are implemented within a single web‐based workflow: subcellular context from compartment‐organized interaction networks, mechanistic context from pathway and kinase–substrate analysis, and regulatory context from isoform and PTM modification‐fraction modules, all coupled to a continuous evidence chain that links each biological summary back to the underlying proteins, peptides, phosphosites, and sample‐level measurements. Rather than replacing established frameworks, ProteoAnalyst connects these analytical layers within a unified workflow, reducing the file conversions, repeated annotations, and tool switching that otherwise fragment interpretation.

Our case studies illustrate how ProteoAnalyst supports exploratory interpretation and hypothesis generation across global proteomics and phosphoproteomics datasets. Its network modules allow users to weigh data‐driven co‐expression against curated protein interactions and subcellular localization, which helps distinguish meaningful biological relationships from spurious correlations, while the phosphoproteomics workflow links site‐level changes to kinase enrichment and kinase‐substrate relationships. The peptide‐level analyses extend this evidence layer by screening for shifts in isoform usage and relative modification fraction when the required precursor‐level information is available. Dedicated isoform tools address a related question from a different angle: IsoPepTracker (Mahmud & Huang, 2026), for example, identifies the peptides that distinguish canonical and novel isoforms or alternative‐splicing events and are theoretically detectable by shotgun proteomics. Finally, the biomarker module supports candidate discovery and internal performance evaluation, although external validation remains essential prior to clinical translation.

ProteoAnalyst currently focuses on label‐free global proteomics and phosphoproteomics and does not yet natively support labeling‐based workflows such as TMT/iTRAQ and SILAC. However, a processed quantitative matrix derived from a labeled study is, however, a valid input, as in the phosphoproteomics case study. Protein isoform and relative modification‐fraction analyses require suitable precursor‐level evidence, and their outputs should be interpreted as hypothesis generating screens rather than complete isoform or PTM stoichiometry characterizations. Future development will focus on expanding native quantification support, enhancing integration with 3D protein structural information, and extending the platform toward single‐cell proteomics and multi‐omics interpretation.

## AUTHOR CONTRIBUTIONS

**Guangyan Zhou:** Software; writing – review and editing; writing – original draft; data curation; visualization; methodology; investigation. **Gordon Li:** Data curation; resources; methodology; software. **Danielle M. Simons:** Investigation; validation; writing – review and editing; formal analysis. **Alyssa Weinrauch:** Validation; resources; writing – review and editing. **Nolan Sammut:** Resources; validation. **Jelena Kolic:** Resources; writing – review and editing. **Haoning Howard Cen:** Resources; writing – review and editing. **Daina Avizonis:** Writing – review and editing; resources; data curation. **Peter Siegel:** Conceptualization; resources; funding acquisition. **Francis C. Lynn:** Resources; writing – review and editing. **Patrick E. MacDonald:** Resources; funding acquisition; writing – review and editing. **James D. Johnson:** Writing – review and editing; resources; validation; funding acquisition. **Jean‐François Trempe:** Validation; formal analysis; writing – review and editing; investigation; resources; funding acquisition; supervision. **Jianguo Xia:** Funding acquisition; resources; software; validation; conceptualization; visualization; writing – original draft; writing – review and editing; formal analysis; supervision; investigation; methodology; project administration.

## CONFLICT OF INTEREST STATEMENT

J.X. is the founder of XiaLab Analytics, a startup that provides training, maintenance, and long‐term support for omics tools developed by XiaLab. The remaining authors declare no conflicts of interest.

## Supporting information


**Table S1:** Feature comparison of ProteoAnalyst with representative downstream proteomics analysis tools (overview). Detailed panels with per‐tool descriptions and sources follow as. (a) Platform type and accepted inputs. (b) Core quantitative analysis. (c) Functional and network analysis. (d) PTM and peptide‐level evidence. (e) Biomarker and machine‐learning analysis.
**Table S2:** Practical guidance for selecting transformation methods in ProteoAnalyst.
**Table S3:** External reference database and annotation resource versions and download dates. Snapshot dates are the build/download dates of the resource deployed with the released application.
**Table S4:** Benchmark overview.
**Table S5:** (a) iPRG‐2015 (DDA/MaxQuant): ProteoAnalyst (DEqMS method) versus MSstats and msqrob2 over the six pairwise contrasts. msqrob2 and limma (fallback) columns are included for reference. (b) DDA spike‐in benchmark (Cox & Mann 2014 MaxLFQ proteome benchmark, PRIDE PXD000279): HeLa background with *E. coli* spiked at 10 fmol versus 30 fmol.
**Table S6:** (a) LFQbench HYE124 (DIA/Spectronaut) known‐ratio benchmark on the common protein set (*n* = 4511; 2728 species‐defined true positives, 1783 unchanged human proteins). (b) UPS1 example dataset (ExperimentHub EH7782; MassIVE MSV000087597, Spectronaut) benchmark results.
**Table S7:** iPRG‐2015 missing‐value robustness (Tukey's median polish). Additional missing‐completely‐at‐random (MCAR) values were injected into the normalized peptide matrix at increasing rates (mean of three seeds), then summarization and limma differential expression were re‐run and re‐scored against ground truth.
**Table S8:** MSstatsPTM simulations (config 5: 2 groups × 5 replicates; ground truth = per‐site differential status after protein adjustment, 250 differential of 1000 sites). ProteoAnalyst's protein‐adjusted delta method versus MSstatsPTM. Note: significance = protein‐adjusted BH FDR < 0.05. Reproduces end‐to‐end from benchmark_msstatsptm.zip.
**Table S9:** Hogrebe two‐species phosphoproteomics spike‐in (PRIDE PXD007145): ProteoAnalyst default versus MSstatsPTM package.
**Table S10:** iPRG‐2015 (DDA/MaxQuant) differential‐expression runtime versus number of proteins (single core, six pairwise contrasts).
**Table S11:** Isoform‐usage analysis assessed on a controlled two‐isoform in silico mixture.
**Table S12:** Orthogonal transcript evaluation of the isoform‐usage analysis (DM1, PXD025589/E‐MTAB‐10842). ProteoAnalyst proteotypic isoform‐usage effect vs. matched transcript splice‐event ratios (deposit: validation_isoform_transcript_dm1.zip).
**Table S13:** PTM relative modification‐fraction estimator evaluation on a semi‐synthetic mixture with known modification fractions. Known per‐condition oxidation modification fraction imposed on real Case‐Study‐4 signal (deposit: validation_mixture_designs.zip).

## Data Availability

The data that support the findings of this study are available in Zenodo at https://doi.org/10.5281/zenodo.22260116, reference number 22260116. The deposit holds the analysis backend, all benchmark and case‐study inputs, scoring scripts, checksums and session information. It also includes the full palmitate, FB231 and Case Study 4 (isoform/PTM modification‐fraction) matrices. These data were derived from the following resources available in the public domain: iPRG‐2015 controlled‐mixture benchmark: MassIVE RMSV000000249.2 (https://massive.ucsd.edu). LFQbench HYE124 benchmark: MassIVE RMSV000000250.2 (https://massive.ucsd.edu). Two‐species phosphoproteomics spike‐in: PRIDE PXD007145 (https://www.ebi.ac.uk/pride/archive/projects/PXD007145). MaxLFQ two‐proteome DDA spike‐in: PRIDE PXD000279 (https://www.ebi.ac.uk/pride/archive/projects/PXD000279). UPS1‐in‐E. coli DIA spike‐in: MassIVE MSV000087597 (https://massive.ucsd.edu/ProteoSAFe/dataset.jsp?accession=MSV000087597). MSstatsPTM benchmark simulations: https://github.com/devonjkohler/MsstatsPTM_simulations. Phosphoproteomics case study: PRIDE PXD005536 (https://www.ebi.ac.uk/pride/archive/projects/PXD005536). Isoform‐usage validation (proteomics): ProteomeXchange PXD025589 (https://proteomecentral.proteomexchange.org/cgi/GetDataset?ID=PXD025589). Isoform‐usage validation (matched transcriptomics): ArrayExpress E‐MTAB‐10842 (https://www.ebi.ac.uk/biostudies/arrayexpress/studies/E-MTAB-10842).
