# Supplementary material for: ProteoAnalyst: Connecting biological context and peptide‐level evidence in quantitative proteomics and phosphoproteomics
Source: Protein Sci. 2026 Nov 12;35(12):e70851. doi: 10.1002/pro.70851 (PMC13756839; doi:10.1002/pro.70851)
Supplement: Supplementary file 1 [file PRO-35-e70851-s001.docx]

**Supporting Information**

**ProteoAnalyst: connecting biological context and peptide-level evidence in quantitative proteomics and phosphoproteomics**

Guangyan Zhou^1,2^, Gordon Li^1,2^, Danielle M. Simons^3^, Alyssa Weinrauch^4,5^, Nolan Sammut^4^, Jelena Kolic^4,6^, Haoning Howard Cen^4^, Daina Avizonis^7,8^, Peter Siegel^7,8^, Francis C. Lynn^5,9^, Patrick E. MacDonald ^10,11^, James D. Johnson^4,5^, Jean-François Trempe^3,12^, Jianguo Xia^1,2*^

^1^ Department of Microbiology and Immunology, McGill University, Montreal, Quebec, Canada

^2^ Institute of Parasitology, McGill University, Montreal, Quebec, Canada

^3^ Department of Pharmacology and Therapeutics and Centre de Recherche en Biologie Structurale, McGill University, Montreal, Quebec, Canada

^4^ Life Sciences Institute, Department of Cellular and Physiological Sciences & Department of Surgery, University of British Columbia, Vancouver, BC, Canada

^5^ Department of Surgery, University of British Columbia, Vancouver, BC, Canada

^6^ Department of Physiology, University of Alberta, Edmonton, AB, Canada

^7^ Department of Biochemistry, McGill University, Montreal, Quebec, Canada

^8^ Rosalind and Morris Goodman Cancer Institute, McGill University, Montreal, Quebec, Canada

^9^ Diabetes Research Group, BC Children’s Hospital Research Institute, Vancouver, BC, Canada

^10^ Department of Pharmacology, Faculty of Medicine and Dentistry, University of Alberta, Edmonton, AB, Canada

^11^ Alberta Diabetes Institute, University of Alberta, Edmonton, AB, Canada

^12^ Brain Repair and Integrative Neuroscience (BRaIN), Research Institute of the McGill University Health Centre (RI-MUHC), Montréal, Québec, Canada

*Correspondence: [jeff.xia@mcgill.ca](mailto:jeff.xia@mcgill.ca)

# Supplementary Methods

This section describes the analytical methods and default parameters underlying results reported in the main text, provided in response to reviewer requests for additional detail. Parameters are given as their default values; alternatives selectable in the interface are noted where relevant.

## 1. KNN and Bayesian PCA imputation

The K-nearest-neighbours option is a wrapper of the established impute.knn (impute package). It is one of the protein-level missing-at-random methods available at the Normalization step. It replaces each missing value with the unweighted mean of the corresponding values in the k most similar feature rows, where similarity is Euclidean distance computed over the samples in which the target feature is observed. Inherited impute.knn defaults apply: features missing in over 50% of samples receive the per-sample mean, any sample over 80% missing raises an error, and matrices above 1,500 features are partitioned by two-means clustering before neighbour search.

Missing values can be imputed by Bayesian principal component analysis (BPCA), implemented in the pcaMethods package. BPCA fits a low-rank model of the abundance matrix by variational Bayes, with an automatic relevance determination prior that suppresses unsupported components, and replaces each missing entry with its posterior expectation. ProteoAnalyst runs BPCA on the log-transformed matrix with five principal components and mean centering.

## 2. Top-N peptide summarization.

Under Top-N (Hi3/Top3) option, each protein is quantified from its N most intense proteotypic peptides, where N defaults to 3. Peptide rank is determined once per protein from the mean intensity across all samples, so the same N peptides are used in every sample. The selected peptides' intensities are averaged per sample on the linear scale and returned to log2. Proteins with fewer than N observed peptides are quantified from those available.

## 3. DEqMS and limma fallback

DEqMS extends the limma model by making each feature's prior residual variance a function of the number of peptides or peptide-spectrum matches (PSMs) supporting it, so that estimates backed by more evidence receive tighter variances; it is the default for inputs that carry such counts (for example, DDA searches). The count is the number of peptides or PSMs quantifying each protein, taken directly from the quantification output.

Count validity is evaluated before fitting. DEqMS is applied when at least three features carry finite positive counts and at least three distinct count values are present; once this check passes, any remaining missing or non-positive counts are set to one, following DEqMS's minimum-count convention. If the counts are unavailable or fail this check, or if the DEqMS variance-moderation (spectraCounteBayes) step fails, the standard limma moderated t-test (eBayes) on the same design and contrasts is returned instead. The method actually used and the count source are recorded in the analysis report.

## 4. Peptide-level isoform-usage analysis

The isoform-usage detection is done in the following steps: First, precursors are filtered to proteotypic peptides that are present in the normalized peptide-level matrix and carry a valid protein assignment. Second, the grouping level is auto-detected: if a sufficient fraction of protein identifiers carry UniProt isoform suffixes (e.g. Q09028-2, isoform 2 of Q09028), isoforms are grouped under their canonical protein; otherwise they are grouped by gene. Third, each isoform must be supported by at least two distinct proteotypic peptide sequences, with charge states and modified forms of the same sequence counted once, and quantified in at least three samples; its per-sample abundance is taken as the mean of its precursors. The output reports both the number of distinct peptide sequences (Peptides.A/B) and the number of precursors (Precursors.A/B) supporting each isoform. Fourth, a group is retained only if at least two of its isoforms satisfy both requirements, so the two-isoform minimum is applied to isoforms that survive filtering rather than to all annotated isoforms. Fifth, a per-group omnibus test compares nested linear models by analysis of variance, in which a sample term absorbs overall gene or protein abundance and a condition-by-isoform interaction term tests for condition-specific shifts in isoform composition. Sixth, for each isoform pair, the per-sample log-ratios and the group's total abundance are each modeled with limma. An isoform change is significant when the omnibus and ratio false discovery rates are both below 0.05 and the total group abundance is not itself significantly changed (unadjusted p > 0.1).

## 5. Enrichment analysis: over-representation and GSEA

Over-representation analysis uses a one-sided hypergeometric test, evaluating the probability of observing at least as many members of an annotation set among the selected features as were found. The background universe defaults to the measured features of the uploaded dataset; users can switch it to the full gene set library. Annotation sets are restricted to the measured universe, so the tested population and the reported set sizes refer to the same pool. Raw p-values are adjusted across all tested sets by the Benjamini-Hochberg method; the adjustment is computed before the display filter that hides sets with fewer than three query hits, so filtering does not alter the reported false discovery rates.

Gene set enrichment analysis is run with the fgsea R package on all mapped measured features, ranked by fold change (the default) or by the moderated t-statistic. Annotation sets are restricted to between 5 and 500 members and scored in both the positive and negative direction. Enrichment p-values are estimated with fgsea's multilevel (adaptive Monte Carlo) procedure at its default settings. Because this procedure is stochastic, a fixed seed is set before every call, so repeated runs on the same input return identical results. Each gene set is reported with its normalized enrichment score (NES), whose sign gives the direction of enrichment along the ranking and whose magnitude gives its strength, and its Benjamini-Hochberg adjusted p-value. Database versions and download dates are listed in Table S3.

## 6. Phosphosite correction by parent-protein abundance

This function aims to distinguish genuine changes in site-level modification from changes driven by parent-protein abundance. For each phosphosite with an unambiguously matched parent protein quantified in the same samples, a protein-adjusted value is formed per sample as log2(site) − log2(parent). Sites without an eligible matched parent, or lacking overlapping samples, are dropped rather than reported unadjusted; because the parent signal is subtracted within each sample, the site and its parent must be measured in the same samples. The protein-adjusted matrix then enters the platform's standard differential expression analysis.

# Supplementary Tables

Table S1. Feature comparison of ProteoAnalyst with representative downstream proteomics analysis tools (overview). Detailed panels with per-tool descriptions and sources follow as Tables S1a–S1f.

| **Capability** | **ProteoAnalyst** | **Perseus** | **MSstats / MSstatsPTM** | **Monash Analyst Suite** | **ProteoArk** |
| --- | --- | --- | --- | --- | --- |
| **Deployment and access** | | | | | |
| Platform type | Web platform | Desktop | R / Bioconductor (+Shiny) | Web applications | Web platform |
| Browser-based, no installation | ✓ | — | ◐ | ✓ | ✓ |
| Free for academic use | ✓ | ✓ | ✓ | ✓ | ✓ |
| **Data handling** | | | | | |
| Multi-tool input support (MaxQuant, FragPipe/MSFragger, DIA-NN, Spectronaut) | ✓ | ◐ | ✓ | ✓ | ✓ |
| Quality control and normalization | ✓ | ✓ | ✓ | ✓ | ✓ |
| Differential expression analysis | ✓ | ✓ | ✓ | ✓ | ✓ |
| **Feature- and evidence-level resolution** | | | | | |
| Peptide- and precursor-level analysis | ✓ | ◐ | ✓ | ◐ | — |
| Isoform-usage analysis | ✓† | — | — | — | — |
| Protein-abundance-corrected PTM site analysis | ✓ | ◐ | ✓ | ◐ | — |
| Evidence traceability to underlying measurements | ✓ | ◐ | ◐ | ◐ | ◐ |
| **Functional interpretation** | | | | | |
| Functional enrichment (GO / pathway) | ✓ | ✓ | — | ✓ | ✓ |
| Pathway visualization | ✓ | — | — | ✓ | ◐ |
| Protein–protein interaction (PPI) network analysis | ✓ | ◐ | ◐ | ✓ | ✓ |
| Co-expression network analysis | ✓ | ◐ | — | — | — |
| Subcellular-compartment network context | ✓ | — | — | — | — |
| **Specialized workflows** | | | | | |
| Phosphoproteomics workflow | ✓ | ✓ | ✓ | ✓ | — |
| Kinase enrichment / kinase–substrate network | ✓ | ✓ | — | ✓ | — |
| Biomarker / machine-learning evaluation | ✓ | ✓ | — | — | — |

**Legend:** ✓ supported in the standard distribution; ◐ partial, indirect, or extension-/plugin-dependent; — not identified in the cited sources. † For precursor-level DIA-NN uploads. The Monash Analyst Suite column represents the suite collectively: a capability provided by any of its applications (LFQ-, DIA-, TMT-, Phospho-, FragPipe- or Pathway-Analyst) is scored as supported, including pathway diagrams and STRING networks, which are provided by the separate Pathway-Analyst app from results exported by the other applications. How these capabilities are organized across applications is described in the main text and in Tables S1a–S1f.

Note:

Entries reflect the documented capabilities of each tool's standard distribution, based on its primary publications and user documentation as of June 2026: Perseus (5); MSstats and MSstatsPTM (6, 47); the Monash Analyst Suite, including LFQ-Analyst, FragPipe-Analyst and Phospho-Analyst (9, 10, 68); and ProteoArk (12). Symbols indicate whether a capability is present, not its depth, performance, or relative quality, and several tools can be extended through scripting or third-party plugins beyond what is captured here. “Not identified in the cited sources” is not proof that a feature is absent from every version, plug-in, or workflow. The comparison is restricted to label-free global proteomics and phosphoproteomics, the scope shared by all listed tools; labeling-based workflows lie outside this scope.

**S1a**. Platform type and accepted inputs

| **Tool** | **Platform** | **Accepted inputs** |
| --- | --- | --- |
| **ProteoAnalyst** | Web (Java/Jakarta EE front end with an R backend) | MaxQuant (proteinGroups.txt, peptides.txt/evidence.txt, Phospho(STY)Sites.txt); FragPipe (combined_protein/peptide/ion.tsv); DIA-NN (pg_matrix.tsv, report.pr_matrix.tsv, report.tsv); Spectronaut protein and peptide/precursor tabular reports; generic protein/peptide tables. Available downstream functions depend on the uploaded data level and annotations |
| **Perseus** | Desktop application (Windows) | MaxQuant tables (protein groups, peptides, Phospho(STY) sites) and generic tab-separated matrices |
| **MSstats family** | R/Bioconductor packages; MSstatsShiny point-and-click GUI | MSstats-format long tables. MSstatsConvert lists converters for DIA-Umpire, MaxQuant, OpenMS, OpenSWATH, Progenesis, Proteome Discoverer, Skyline, SpectroMine, Spectronaut, Philosopher, DIA-NN, FragPipe and MetaMorpheus; TMT workflows use MSstatsTMT. Converter coverage differs among family packages |
| **Monash Analyst Suite** | Web (R/Shiny), one app per upstream workflow | **LFQ-Analyst**: MaxQuant proteinGroups plus a design file; **Phospho-Analyst**: MaxQuant Phospho(STY)Sites and optionally proteinGroups; **DIA-Analyst:** Spectronaut or DIA-NN protein matrices; **TMT-Analyst:** Proteome Discoverer protein-level output. **FragPipe-Analyst**: FragPipe DDA protein and peptide reports, TMT gene/protein/peptide and single-site reports, and documented DIA protein-, peptide- and site-level reports. |
| **ProteoArk** | Web (Django) plus Docker standalone | Proteome Discoverer, MaxQuant, MSFragger outputs (label-free and SILAC/iTRAQ/TMT); generic normalized CSV |

## S1b. Core quantitative analysis

| **Tool** | **QC / normalization / imputation** | **Differential expression** |
| --- | --- | --- |
| **ProteoAnalyst** | QC plots before and after processing; log2 (median-centred by default), VSN or quantile normalization, with RLR and LOESS in the phosphoproteomics workflow; percentage-based missing-value filtering; MNAR imputation (limit-of-detection  Replacement by default) and ten MAR methods (KNN by default); optional imputation using MBimpute during summarization. | limma; optional DEqMS count-dependent variance step with limma fallback; msqrob2; multi-factor/covariate designs |
| **Perseus** | Free-form transform (log2 by default) and eleven normalization activities; count-based valid-value filtering; imputation from a downshifted normal distribution or by a constant, extensible through plugins. | t-tests/ANOVA with permutation-based FDR |
| **MSstats family** | Model-based processing (MSstats dataProcess): equalize-medians (default), quantile or global-standards normalization; Tukey median polish or linear-model summarization; top-N feature selection; censored intensities imputed by an accelerated-failure-time model. | Linear mixed-effects models (DDA/DIA/SRM); MSstatsTMT |
| **Monash Analyst Suite** | Per-app QC plots. Normalization varies by application: VSN, median, median subtraction or internal reference scaling. Nine imputation methods (Perseus-type downshift by default), five in FragPipe-Analyst. Filtering is fixed in LFQ-Analyst and percentage-based in FragPipe-Analyst. | limma ; Benjamini–Hochberg and t-statistic (fdrtool) FDR options; paired designs are documented in LFQ-/Phospho-Analyst, and multifactor metadata in DIA-/TMT-Analyst |
| **ProteoArk** | Median, sum, quantile (default), internal reference scaling, Z-score or trimmed-mean normalization; imputation by a constant, one fifth of the lowest value, or missForest; a fixed missing-value rule; optional ComBat or limma batch correction. | Welch's t-test (default), two-sample t-test, one- or two-way ANOVA, or limma; Bonferroni, Benjamini-Hochberg or Benjamini-Yekutieli adjustment; pairwise control-versus-test contrasts. |

## S1c. Functional and network analysis

| **Tool** | **Enrichment** | **Networks** |
| --- | --- | --- |
| **ProteoAnalyst** | Hypergeometric ORA, and GSEA by fgsea, over GO, KEGG, Reactome, PANTHER GO-Slim, subcellular-compartment and motif libraries with organism-dependent coverage. | PPI from STRING (11 organisms selectable), InnateDB, iRefIndex, and human-only IntAct, HuRI, Rolland and OmniPath. Minimum-network and prize-collecting Steiner forest; nine layouts including a subcellular-compartment view, a 3D view and community detection; CEMiTool co-expression modules. |
| **Perseus** | Fisher exact test on categorical columns, plus 1D and 2D annotation enrichment (Mann-Whitney based). Annotation files are downloaded and installed by the user. | PerseusNet imports user-supplied edge tables, GraphML, SIF or JSON. AP-MS network generation from volcano analysis, degree and scale-free statistics, and an interactive node-link view. |
| **MSstats family** | - not identified in cited sources | Through the companion MSstatsBioNet: literature-derived interaction statements fetched live, filterable by source, evidence count and curation, drawn in a hierarchical layout. |
| **Monash Analyst Suite** | ORA through Enrichr against fixed human libraries in most apps, and clusterProfiler in FragPipe-Analyst. The separate Pathway-Analyst app adds g:Profiler, fgsea GSEA and a user-supplied background. It also provides KEGG pathway diagrams. | Provided by the separate Pathway-Analyst app: significant genes mapped to STRING and drawn with selectable confidence, igraph module clustering, a downloadable interaction table and a link out to STRING. |
| **ProteoArk** | ORA delegated to the g:Profiler API, covering GO, KEGG, Reactome, WikiPathways and further sources, with g:SCS correction. | STRING, through the embedded STRING widget with user-selected confidence, plus a downloadable interaction-partner table. |

## S1d. PTM and peptide-level evidence

| **Tool** | **Phospho / PTM** | **Peptide-level drill-down & isoform** |
| --- | --- | --- |
| **ProteoAnalyst** | Site-level differential analysis by limma or msqrob2; protein-adjusted site change by a limma delta method; relative modification-fraction analysis (modified/(modified + unmodified)) for DIA-NN precursor matrices; kinase over-representation with signed KSEA scores and kinase-substrate networks; predefined sequence-motif classes. | Peptide and precursor input from MaxQuant, FragPipe, DIA-NN and Spectronaut, with seven summarization methods. A feature dialog traces protein to constituent peptides with per-sample plots, from the volcano, Manhattan, pathway, network and result-table views; isoform-usage testing for DIA-NN input. |
| **Perseus** | MaxQuant site tables, PhosphoSitePlus and linear motif annotation, kinase-substrate relations and KSEA. Site statistics use the generic t-test and ANOVA engine. | Peptide, precursor and site tables load as independent matrices through the same generic matrix upload as protein groups. Protein quantities are inherited from the search engine. |
| **MSstats family** | MSstatsPTM fits site and unmodified-protein models separately and reports their difference with combined standard errors, as a relative protein-adjusted change. | Peptide and precursor level is the native modelling unit, with differential analysis reported at protein level. MSstatsShiny plots feature-level profiles for a selected protein, with feature-level export. |
| **Monash Analyst Suite** | Phospho-Analyst: limma site-level differential expression with a localization-probability filter; parent-protein adjustment as a per-condition scaling factor, uncertainty-propagated adjustment; KSEA kinase-substrate enrichment. | FragPipe-Analyst: peptide-level and site-level input. Phospho-Analyst: compares site and protein results. TMT-Analyst: single-peptide filter. |
| **ProteoArk** | - not identified in cited sources | Peptide-level input in the label-free branch, collapsed to protein-level iBAQ, TOP3 or NSAF using in-silico tryptic peptide counts, and returned as a downloadable table. |

## S1e. Biomarker and machine-learning analysis

| **Tool** | **Biomarker / ML** |
| --- | --- |
| **ProteoAnalyst** | Univariate ROC and exploratory multivariate models (elastic net, random forest, linear SVM, PLS-DA, logistic regression); |
| **Perseus** | Classification with cross-validation and prediction, feature and parameter optimization, and performance-curve activities |
| **MSstats family** | - not identified in cited sources |
| **Monash Analyst Suite** | - not identified in cited sources |
| **ProteoArk** | - not identified in cited sources |

## S1f. Reproducibility and openness

| **Tool** | **Openness / replay** |
| --- | --- |
| **ProteoAnalyst** | Public MIT-licensed R source backend; GUI exports (Rhistory plus input/output tables) support auditing and reconstruction. |
| **Perseus** | Free desktop application; session files capture analyses; plugin API |
| **MSstats family** | Open-source, versioned Bioconductor packages; MSstatsShiny for GUI reproducibility |
| **Monash Analyst Suite** | Open-source R/Shiny apps on GitHub; |
| **ProteoArk** | Open source (GitHub); Docker standalone for local execution |

**Table S2. Practical guidance for selecting transformation methods in ProteoAnalyst**

| **Method** | **Best suited for** | **Diagnostic cue** | **Cautions** |
| --- | --- | --- | --- |
| **Log2 transformation** | Positive raw intensity data with right-skewed abundance distributions or multiplicative technical variation. | Density plots or boxplots are strongly right-skewed, and high-intensity features dominate the scale. | Does not correct sample-wide shifts and should not be applied to data that are already log-transformed or contain many non-positive values. |
| **Log2 transformation + median centering** | Positive raw intensity data that need log scaling and sample-level median alignment. | After log scaling, boxplots show similar distributions but shifted medians. | Assumes most proteins are stable globally and may be inadequate when bias varies with intensity. |
| **Quantile normalization** | Datasets where technical effects are expected to alter intensity distributions across samples. | Density distributions differ broadly across the full abundance range. | Avoid when broad biological shifts are expected, because they may be compressed as if they were noise. |
| **Variance Stabilizing Normalization (VSN)** | Datasets where variance depends strongly on signal intensity. | Mean-variance plots show stronger variability at specific intensity ranges. | Can be more aggressive than simple scaling. |

**Table S3.** External reference database and annotation resource versions and download dates. Snapshot dates are the build/download dates of the resource deployed with the released application.

| **Resource** | **Role** | **Source / version** | **Snapshot / download date** |
| --- | --- | --- | --- |
| **Gene/protein ID mapping (<org>_genes.sqlite, 130+ organisms)** | Identifier annotation and cross-mapping (UniProt, Entrez, RefSeq, Ensembl, symbol) | NCBI Gene + UniProt | 2023-12-15 |
| **GO BP / CC / MF gene sets** | Over-representation analysis, GSEA | org.Hs.eg.db + GO.db 3.22.0 (Bioconductor) | 2025-07-22 |
| **Reactome gene sets** | Over-representation analysis, GSEA | reactome.db 1.95.0 (Bioconductor) | 2026-01-06 |
| **KEGG pathways** | Over-representation analysis, GSEA, pathway networks | KEGGREST; KEGG release of 2026-08-20 | 2026-08-20 |
| **STRING** | Protein-protein interaction networks | STRING v12.0 (combined score >= 900 by default, UI-selectable 401-999) | 2025-12-28 |
| **IntAct** | Protein-protein interaction networks | IntAct Release 247 | 2025-11-24 |
| **HuRI** | Protein-protein interaction networks | HI-union (2020) | 2025-11-24 |
| **InnateDB (IMEx Interactome)** | Protein-protein interaction networks | IMEx-curated; no pinned version string | 2022-01-29 (InnateDB all.mitab.gz release) |
| **OmniPath** | PPI networks; TF regulons (DoRothEA) | OmniPath | 2025-11-24 |
| **Kinase-substrate sets: GPS, NetworKIN, PhosphoNetworks, RegPhos** | Kinase substrate-set enrichment and KSEA (per organism: hsa, mmu, rno, sce, spo, cel, dme, ath) | GPS 5.0; NetworKIN; PhosphoNetworks; RegPhos 2.0 | 2025-12-12 |
| **TF-gene regulation** | TF-target networks | ENCODE / TRRUST / ChEA | 2024-06-20 |

**Table S4. Benchmark overview.**

| **Table** | **Dataset (accession)** | **Mode** | **Ground truth** | **Primary readout** | **Deposit bundle** |
| --- | --- | --- | --- | --- | --- |
| S5a | iPRG-2015 (MassIVE RMSV000000249.2) | DDA / MaxQuant | Six spiked proteins (Latin square); yeast background null | Sens., spec., empirical FDR, AUC | benchmark_maxquant_iprg2015 |
| S5b | MaxLFQ proteome benchmark (PRIDE PXD000279) | DDA / MaxQuant | E. coli 10 vs 30 fmol in constant HeLa | ROC AUC, fold-change separation | benchmark_maxquant_deqms_pxd000279 |
| S6a | LFQbench HYE124 (Navarro 2016) | DIA / Spectronaut | Known human:yeast:E. coli ratios | Sens., spec., empirical FDR, AUC, FC correlation | benchmark_spectronaut_navarro2016 |
| S6b | UPS1 example dataset (ExperimentHub EH7782; MSV000087597) | DIA / Spectronaut | 48 UPS1 standards in constant E. coli background | Sens., spec., empirical FDR, AUC | benchmark_ups1_dia_deqms |
| S7 | iPRG-2015 with injected MCAR missingness | DDA / MaxQuant | As S4 | Metric degradation vs missing rate | benchmark_maxquant_iprg2015 |
| S8 | MSstatsPTM simulations (config 5) | Simulated PTM | Per-site differential status (250 of 1,000) | Sensitivity, specificity, emp. FDR, ROC AUC | benchmark_msstatsptm |
| S9 | Hogrebe phospho spike-in (PRIDE PXD007145) | DDA phospho / MaxQuant | Species membership; spike ratios 1:4:10 | ROC AUC, yeast median log2FC | benchmark_hogrebe_phospho |
| S10 | iPRG-2015 runtime sweep | DDA / MaxQuant | n/a (wall-clock timing) | DE runtime vs protein count | benchmark_maxquant_iprg2015 |

**Table S5a.** iPRG-2015 (DDA/MaxQuant): ProteoAnalyst (DEqMS method) versus MSstats and msqrob2 over the six pairwise contrasts. msqrob2 and limma (fallback) columns are included for reference.

|  | **ProteoAnalyst** | **MSstats** | **msqrob2** | **limma** |
| --- | --- | --- | --- | --- |
| **Sensitivity (spike-ins recovered, correct sign, adj. p < 0.05)** | 0.87 | 0.87 | 0.83 | 0.80 |
| **Empirical FDR (nominal 5%)** | 0.00 | 0.21 | 0.04 | 0.04 |
| **Specificity** | >0.9999 | 0.9996 | 0.9999 | 0.9999 |
| **Fold-change accuracy vs truth (Pearson r)** | 0.97 | 0.98 | 0.97 | 0.97 |
| **Fold-change RMSE (log2)** | 0.95 | 0.80 | 0.91 | - |
| **ROC AUC (by p-value)** | 0.98 | 0.96 | 0.98 | 0.97 |
| **False positives (of 16,536 true-negative tests)** | 0 | 7 | 1 | 1 |
| **Background mean log2FC (bias; target 0)** | −0.04 | - | −0.04 | - |

ABRF iPRG-2015 spike-in (MassIVE RMSV000000249.2): constant S. cerevisiae background (true log2FC = 0, true negatives) with six proteins spiked in a Latin square across 4 conditions × 3 replicates (2,877 proteins × 12 samples), giving known differential status and true log2FC in all six pairwise contrasts.

**Table S5b.** DDA spike-in benchmark (Cox & Mann 2014 MaxLFQ proteome benchmark, PRIDE PXD000279): HeLa background with E. coli spiked at 10 vs 30 fmol.

|  | **limma** | **DEqMS** | **msqrob2** | **MSstats** | **DEqMS native (ref)** |
| --- | --- | --- | --- | --- | --- |
| **ROC AUC (by p-value)** | 0.839 | 0.810 | 0.848 | 0.784 | 0.813 |
| **ROC AUC (by \|log2FC\|)** | 0.890 | 0.890 | 0.894 | 0.881 | 0.909 |
| **Fold-change separation, E. coli-human (expected 1.585)** | 1.74 | 1.74 | 1.74 | 2.00 | 1.74 |
| **E. coli in top 100** | 94 | 96 | 93 | 85 | 96 |

Expected E. coli log2FC = log2(3) = 1.585; human 1:1. Because the asymmetric spike shifts overall composition, scoring uses metrics insensitive to global intensity shifts (species ROC AUC and fold-change separation) on the n = 4,311 proteins quantified in common. Configurations are described in **Supplementary Text 1**. Reproduces end-to-end from benchmark_maxquant_deqms_pxd000279.zip.

**Table S6a.** LFQbench HYE124 (DIA/Spectronaut) known-ratio benchmark on the common protein set (n = 4,511; 2,728 species-defined true positives, 1,783 unchanged human proteins).

| **Metric** | **ProteoAnalyst (msqrob2 default)** | **limma** | **DEqMS** | **MSstats (reference)** |
| --- | --- | --- | --- | --- |
| **Sensitivity** | 0.891 | 0.880 | 0.886 | 0.901 |
| **Specificity** | 0.847 | 0.809 | 0.781 | 0.907 |
| **Empirical FDR** | 0.100 | 0.124 | 0.138 | 0.063 |
| **FC correlation (r)** | 0.929 | 0.929 | 0.929 | 0.953 |
| **ROC AUC** | 0.937 | 0.923 | 0.926 | 0.965 |

Design and configurations are described in Supplementary Text 2. Reproduces end-to-end from benchmark_spectronaut_navarro2016.zip.

**Table S6b.** UPS1 example dataset (ExperimentHub EH7782; MassIVE MSV000087597, Spectronaut) benchmark results.

| **Contrast (true log2FC)** | **Method** | **Sensitivity** | **Specificity** | **Empirical FDR** | **ROC AUC (by p-value)** |
| --- | --- | --- | --- | --- | --- |
| **5 vs 2.5 fmol (1.00)** | limma | 0.46 | 0.999 | 0.083 | 0.840 |
|  | DEqMS | 0.52 | 0.999 | 0.038 | 0.855 |
|  | msqrob2 | 0.46 | 0.999 | 0.083 | 0.842 |
|  | MSstats | 0.19 | 0.999 | 0.100 | 0.799 |
| **25 vs 5 fmol (2.32)** | limma | 1.00 | 0.996 | 0.111 | 1.000 |
|  | DEqMS | 1.00 | 0.991 | 0.226 | 1.000 |
|  | msqrob2 | 1.00 | 0.996 | 0.111 | 1.000 |
|  | MSstats | 0.98 | 0.987 | 0.329 | 1.000 |
| **10 vs 1 fmol (3.32)** | limma | 0.88 | 0.999 | 0.023 | 0.981 |
|  | DEqMS | 0.92 | 0.992 | 0.214 | 0.983 |
|  | msqrob2 | 0.88 | 0.999 | 0.023 | 0.982 |
|  | MSstats | 0.75 | 0.993 | 0.250 | 0.973 |

Empirical FDR at nominal 0.05; design and configurations are described in **Supplementary Text 2**. Reproduces end-to-end from benchmark_ups1_dia_deqms.zip.

**Table S7.** iPRG-2015 missing-value robustness (Tukey's median polish). Additional missing-completely-at-random (MCAR) values were injected into the normalized peptide matrix at increasing rates (mean of 3 seeds), then summarization and limma differential expression were re-run and re-scored against ground truth.

| **Induced MCAR** | **Total missing** | **Proteins** | **Sensitivity** | **Specificity** | **Empirical FDR** | **Fold-change RMSE (log2)** | **ROC AUC (by p-value)** |
| --- | --- | --- | --- | --- | --- | --- | --- |
| **0%** | 12.1% | 2809 | 0.81 | 0.9999 | 0.065 | 0.93 | 0.972 |
| **5%** | 16.5% | 2746 | 0.84 | 0.9999 | 0.061 | 0.92 | 0.964 |
| **10%** | 20.9% | 2668 | 0.81 | 1.0000 | 0.023 | 1.15 | 0.950 |
| **20%** | 29.7% | 2489 | 0.77 | 0.9997 | 0.135 | 1.27 | 0.958 |
| **30%** | 38.5% | 2291 | 0.81 | 0.9998 | 0.109 | 1.33 | 0.942 |
| **40%** | 47.2% | 2051 | 0.79 | 0.9999 | 0.045 | 1.33 | 0.948 |

**Table S8.** MSstatsPTM simulations (config 5: 2 groups × 5 replicates; ground truth = per-site differential status after protein adjustment, 250 differential of 1,000 sites). ProteoAnalyst's protein-adjusted delta method versus MSstatsPTM. Note: significance = protein-adjusted BH FDR < 0.05. Reproduces end-to-end from benchmark_msstatsptm.zip.

|  | **Method** | **Sites** | **Differential** | **Sensitivity** | **Specificity** | **Emp.FDR** | **ROC AUC** |
| --- | --- | --- | --- | --- | --- | --- | --- |
| **sim1** | **ProteoAnalyst** | 1000 | 250 | 1.00 | 0.987 | 0.038 | 1.000 |
| **sim1** | **MSstatsPTM** | 1000 | 250 | 0.98 | 0.985 | 0.043 | 0.999 |
| **sim2** | **ProteoAnalyst** | 1000 | 250 | 0.94 | 0.989 | 0.033 | 0.997 |
| **sim2** | **MSstatsPTM** | 1000 | 250 | 0.88 | 0.991 | 0.031 | 0.993 |

**Table S9.** Hogrebe two-species phosphoproteomics spike-in (PRIDE PXD007145): ProteoAnalyst default versus MSstatsPTM package

| **Contrast (true log2FC)** | **ROC AUC (ProteoAnalyst)** | **ROC AUC (MSstatsPTM 2.12.0)** | **ProteoAnalyst** **yeast median log2FC** |
| --- | --- | --- | --- |
| **R4-R1 (2.00)** | 0.961 | 0.903 | 1.83 |
| **R10-R1 (3.32)** | 0.974 | 0.877 | 3.01 |
| **R10-R4 (1.32)** | 0.895 | 0.789 | 1.17 |

Design, configurations, and the attribution of the ranking gap are described in **Supplementary Text 5**. Reproduces end-to-end from benchmark_hogrebe_phospho.zip.

**Table S10.** iPRG-2015 (DDA/MaxQuant) differential-expression runtime versus number of proteins (single core, six pairwise contrasts).

| **Proteins** | **limma (s)** | **DEqMS (s)** | **msqrob2 (s)** |
| --- | --- | --- | --- |
| 719 | 0.23 | 10.14 | 8.83 |
| 1438 | 0.29 | 10.39 | 15.11 |
| 2158 | 0.27 | 10.82 | 21.74 |
| 2877 | 0.31 | 11.22 | 28.31 |

**Table S11.** Isoform-usage analysis assessed on a controlled two-isoform in-silico mixture.

| **Metric** | **Value** |
| --- | --- |
| **Calibration** **slope (measured vs imposed ratio)** | 1.004 |
| **Calibration intercept** | 0.001 |
| **Calibration R² / Pearson r** | 0.948 / 0.974 |
| **RMSE (log2)** | 0.35 |
| **Discrimination AUC (switch vs null)** | 0.941 |
| **Power at Δ = 0.5 / 1 / 2 / 3 log2** | 28% / 60% / 80% / 76% |

*Fully known ground-truth isoform ratios (deposit: validation_mixture_designs.zip).*

**Table S12.** Orthogonal transcript evaluation of the isoform-usage analysis (DM1, PXD025589 / E-MTAB-10842). ProteoAnalyst proteotypic isoform-usage effect vs matched transcript splice-event ratios (deposit: validation_isoform_transcript_dm1.zip).

| **Metric** | **Value** |
| --- | --- |
| **Isoform-usage effect vs transcript ratio (Pearson r)** | 0.944 (n = 12) |
| **Sign concordance** | 11 / 12 |
| **Omnibus interaction test significant** | 10 / 12 |
| **Protein-vs-transcript concordance** | r = 0.95 (study) vs 0.944 (ProteoAnalyst) |

**Table S13. PTM relative modification-fraction estimator evaluation** on a semi-synthetic mixture with known modification fractions. Known per-condition oxidation modification fraction imposed on real Case-Study-4 signal (deposit: validation_mixture_designs.zip).

| **Metric** | **Value** |
| --- | --- |
| **Calibration slope (recovered vs imposed)** | 1.010 |
| **Calibration R²** | 0.955 |
| **Modification-fraction recovery (Pearson r)** | 0.963 |
| **Discrimination AUC (changed vs null)** | 0.972 |
| **False-positive rate on null sites (BH FDR < 0.05)** | 1.4% |
| **Abundance-only sites called significant** | 1.9% (naive unadjusted test: 44.9%) |
| **Empirical FDR among calls (vs naive modified-intensity test)** | 2.3% (naive: 43.4%) |
| **Power by \|Δ modification fraction\| (small → large)** | 0.54 / 0.93 / 1.00 / 1.00 |

**Supplementary Text**

**1. Differential expression, DDA (MaxQuant)**

The ABRF iPRG-2015 spike-in study tests whether ProteoAnalyst can recover known differences on data where true positives and true negatives are both fixed by design. ProteoAnalyst uses DEqMS because valid peptide-count information was available, and all methods were scored on the protein set quantified in common with MSstats. ProteoAnalyst can estimate spike-in ratios closely (Pearson r = 0.97; fold-change RMSE 0.95 log2, higher than the MSstats reference at 0.80), and background proteins centered near zero (mean log2 fold change −0.04), consistent with little systematic drift. ProteoAnalyst matched MSstats on sensitivity among quantified spike-ins (0.867 for both) while returning no false positives among 16,536 true-negative tests, against seven for MSstats. The empirical FDR fell from 0.21 to 0.00 at a nominal 0.05 threshold: the two workflows differ in error control rather than in detection, and their effect estimates remained concordant. A second, independent DDA spike-in on DEqMS's own label-free example (the MaxLFQ two-proteome mixture, PXD000279; **Supplementary Table S5b**) gave the same picture: all methods separated the E. coli spike-in from the human background (ROC AUC 0.88–0.91 by fold change on the common protein set), and ProteoAnalyst's DEqMS result reproduced the canonical DEqMS analysis of that dataset. The proteinGroups configurations (limma, DEqMS and msqrob2) ran with a ≥2-observations-per-group filter and no imputation; MSstats ran dataProcess on peptides.txt, its required feature-level input, with the canonical DEqMS vignette pipeline on the same file as an independent reference.

**2. Differential expression, DIA (Spectronaut)**

On the LFQbench HYE124 mixture **(Supplementary Table S6a),** in which human, yeast and E. coli proteins are mixed at A-versus-B ratios of 1:1 (true log2FC = 0; true negatives), 2:1 (+1) and 1:4 (−2), ProteoAnalyst's msqrob2 option recovered species-defined true positives with sensitivity 0.891 at an empirical FDR of 0.100 (nominal 0.05), fold-change correlation r = 0.929, and ROC AUC 0.937 on the common protein set (n = 4,511). The published MSstats reference outperformed ProteoAnalyst's default on all scored metrics (sensitivity 0.901 versus 0.891, specificity 0.907 versus 0.847, empirical FDR 0.063 versus 0.100, fold-change correlation 0.953 versus 0.929, and ROC AUC 0.965 versus 0.937), partly attributable to its design-informed normalization (equalize-medians on the human proteins known to be unchanged). Among ProteoAnalyst's integrated external methods under default data processing, msqrob2 had the best performance (empirical FDR 0.100, versus 0.124 for limma and 0.138 for DEqMS).

A second, independent DIA spike-in on the UPS1 in E. coli dataset, DEqMS's own DIA example (MassIVE MSV000087597; **Supplementary Table S6b**), in which 48 human UPS1 standards are spiked into a constant E. coli background of true negatives (48 true positives per contrast), was consistent across its three contrasts: msqrob2 recovered 0.46, 1.00 and 0.88 of true positives at empirical FDR 0.083, 0.111 and 0.023 (ROC AUC 0.842, 1.000 and 0.982), closely tracking limma. DEqMS was marginally more sensitive at the smallest and largest contrasts (0.52 and 0.92) but showed higher empirical FDR at the two larger contrasts (0.226 and 0.214 versus 0.111 and 0.023), and MSstats was the least sensitive (0.19 at the smallest contrast) with the highest empirical FDR (up to 0.329). The ProteoAnalyst methods shared data processing steps; MSstats ran its own default workflow on the same precursor table.

**3. Robustness to missing values**

Because real experiments rarely match the completeness of a controlled spike-in, we asked how far performance degrades as data are removed. Injecting additional missing values into the iPRG peptide-level data (Tukey summarization with limma differential expression), up to approximately 47% total missingness, left specificity essentially unchanged (≥ 0.99 throughout) and cost little ranking accuracy (ROC AUC 0.97 to 0.95), although the empirical FDR rose as high as 0.14 at intermediate missingness and fold-change accuracy degraded (RMSE 0.93 to 1.33). Sensitivity declined only marginally over the same range (**Supplementary Table S7**).

**4. Protein-adjusted site recovery (simulation)**

We first tested the parent-protein abundance adjustment directly on MSstatsPTM-generated simulations (config 5: two groups × five replicates; 250 truly differential of 1,000 sites, with ground truth defined as per-site differential status after protein adjustment). The MSstatsPTM comparison uses the authors' own published model fits for this configuration (taken from the MSstatsPTM_simulations repository at the pinned commit, not refit); both configurations are scored identically against the same ground-truth table, with Benjamini-Hochberg correction recomputed within contrast. ProteoAnalyst recovered the truly protein-adjusted differential sites with ROC AUC 0.997–1.000 and empirical false-discovery rate at or below the nominal level (0.033–0.038 across replicate simulations; **Supplementary Table S8**). Scored identically on the same simulations, MSstatsPTM reached ROC AUC 0.993–0.999 with empirical false-discovery rate 0.031–0.043; both methods therefore control error at the nominal level, with ProteoAnalyst attaining higher sensitivity (1.00 and 0.94 versus 0.98 and 0.88). Because the simulation encodes the adjusted ground truth, it directly tests the adjustment itself, whereas the real-data spike-in below evaluates real-data phosphosite ranking rather than the adjustment.

**5. Phosphoproteomic accuracy on real data**

The two-species design of Hogrebe et al. (PRIDE PXD007145) provides an external ground truth for site-level phosphoproteomics: yeast phosphopeptides are spiked into a constant human (HeLa) background at ratios 1:4:10 (three replicates per condition; 18,491 MaxQuant Phospho(STY) sites: 3,002 yeast true positives, 15,489 human true nulls), so the truth is independent of any tool's processing. ProteoAnalyst's default workflow separated yeast from human sites with ROC AUC 0.90–0.97, exceeding the native MSstatsPTM package run with label-free defaults on the same site table (AUC 0.79–0.90; essentially unchanged on the full nine runs), and recovered most of the spike fold changes at the largest ratio (median log2 fold change 3.01 against a true 3.32). The gap consists of a difference in differential analysis and a coverage difference, not data processing: on sites common to both tools, ranking by fold change is equally good (AUC 0.95–0.99). On differential analysis, MSstatsPTM's label-free path performs unmoderated per-site tests; a variance-moderated (limma-style) test applied to its own summarized data recovers most of the difference (AUC 0.93/0.95/0.89) and, restricted to the well-observed sites ProteoAnalyst tests, matches ProteoAnalyst (AUC 0.962/0.975/0.905). The rest is coverage policy: MSstatsPTM imputes censored intensities and tests nearly every site, so sparsely observed sites enter the comparison against roughly 15,000 true nulls and add ranking noise, whereas ProteoAnalyst's per-group detection filter excludes them before testing. The threshold-free ranking is therefore the informative comparison **(Supplementary Table S9).**

**6. Runtime and scalability**

Because a web platform must stay responsive at the scale of a typical experiment, we profiled the differential-expression step against protein count on the iPRG-2015 matrix (six pairwise contrasts). At the full size, 2,877 proteins across 12 samples, differential expression took 0.31 s with limma, 11.2 s with DEqMS and 28.3 s with msqrob2. limma and DEqMS scaled sub-linearly with protein count, since fixed setup cost dominates, whereas msqrob2 scaled approximately linearly because it fits peptide-level models for each protein. Data processing is shared across methods and took approximately 56 s so the complete pipeline from native report to differential results ran in approximately 56 s with limma, 68 s with the DEqMS default and 85 s with msqrob2; MSstats on the same data used approximately 160–180 s end to end (**Supplementary Table S10**).

**7. Isoform analysis**

We evaluated the function in two complementary ways. On a controlled two-isoform mixture with known ground truth, it recovered the imposed switches, with detection power rising from 28% at the smallest imposed shift to approximately 80% at larger shifts (**Supplementary Table S11**). On an independent myotonic-dystrophy (DM1) mouse dataset with matched RNA-seq (PXD025589 / E-MTAB-10842), the same omnibus interaction model applied to targeted splice-specific and constitutive PRM peptides was concordant with orthogonal transcript-event changes (Pearson r = 0.944, n = 12; 11 of 12 sign-concordant; **Supplementary Table S12**). This check applies ProteoAnalyst’s interaction model to the study's targeted PRM peptides.

**8. Relative modification-fraction estimator validation**

The relative modification-fraction estimator was evaluated on a semi-synthetic mixture with known modification fractions, constructed by imposing per-condition modification fractions and generating precursor signals from the measured intensities, variances, and missingness of a real DIA dataset (**Supplementary Table S13**). Estimated modification-fraction changes tracked the imposed truth closely (slope 1.01, R2 = 0.96, RMSE 0.045), changed sites were separated from unchanged sites with ROC AUC 0.97 at a controlled empirical false-positive rate (0.014 at BH FDR < 0.05), and detection power rose from 54% at a modification-fraction difference of 0.10 to 93% or higher at 0.20 and above. Sites whose total abundance changed while the modification fraction stayed constant were correctly not called (1.9% at FDR < 0.05), consistent with the test distinguishing modification-fraction change from abundance change.
